# Isolation, Structure Elucidation and Biological Evaluation of Lomaiviticins F–H, Dimeric Benzofluorene Glycosides from Marine-Derived *Micromonospora* sp. Bacterium

**DOI:** 10.3390/md23020065

**Published:** 2025-02-03

**Authors:** Fan Zhang, Wenhui Wang, Doug R. Braun, Gene E. Ananiev, Weiting Liao, Mary Kay Harper, Scott R. Rajski, Tim S. Bugni

**Affiliations:** 1 Department of Pulmonary and Critical Care Medicine, Zhongnan Hospital of Wuhan University, TaiKang Center for Life and Medical Sciences, School of Pharmaceutical Sciences, Key Laboratory of Combinatorial Biosynthesis and Drug Discovery, Ministry of Education, Wuhan University, Wuhan 430071, China; 2022203060042@whu.edu.cn (W.W.); weitingl@whu.edu.cn (W.L.); 2 Pharmaceutical Sciences Division, University of Wisconsin–Madison, Madison, WI 53705, USA; drbraun1@wisc.edu (D.R.B.); scott.rajski@wisc.edu (S.R.R.); tim.bugni@wisc.edu (T.S.B.); 3 Small Molecule Screening & Synthesis Facility, UW Carbone Cancer Center, Madison, WI 53705, USA; geananiev@wisc.edu; 4 Department of Medicinal Chemistry, University of Utah, 30 South 2000 East, Salt Lake City, UT 84112, USA; mk.harper@pharm.utah.edu

**Keywords:** lomaiviticin, benzofluorene, isotopic fine structure, *Micromonospora* sp.

## Abstract

The discovery of new natural products remains a cornerstone of therapeutic innovation, and effective analytical tools for rapid dereplication can significantly accelerate this process. Using Isotopic Fine Structure (IFS) mass spectrometry, we rapidly identified three new dimeric benzofluorene glycosides, lomaiviticins F–H (**1**–**3**), from a marine-derived *Micromonospora* sp. bacterium. These compounds were isolated and structurally elucidated through advanced spectroscopic techniques, including FT-ICR-MS and NMR. Lomaiviticins F–H exhibit unique structural features, notably the 4-*O*-methyl-l-angolosamine moieties, which differentiate them from previously known lomaiviticins A–E. The discovery of these compounds highlights distinct biosynthetic linkages within the lomaiviticin family, particularly the C2–C2′ conjoining bonds characteristic of the dimers. Compounds **1**–**3** were evaluated for in vitro cytotoxicity against a panel of human cancer cell lines; the resulting IC_50_ values confirmed that the dimeric diazofluorenes of lomaiviticins A and B are critical for anticancer activity. These findings emphasize the utility of IFS in expediting natural product discovery while providing valuable insights into structural and functional characterizations of bioactive compounds.

## 1. Introduction

**Figure 1:**
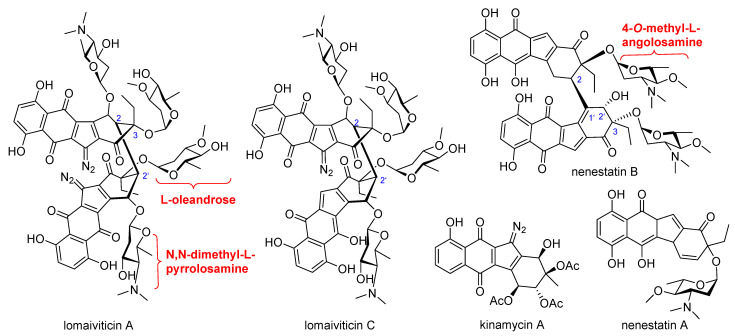
Representative structures of lomaiviticins, kanamycin A, and nenestatins. Lomaiviticins and kinamycins contain diazofluorene functional groups (lomaiviticin A contains two diazofluorenes; lomaiviticin C and kinamycin A contain one diazofluorene), which are unique among known natural products.

Lomaiviticins represent a unique class of natural products derived from marine actinomycetes that have captivated researchers due to their potent anticancer activities [1,2,3,4,5]. Structurally, these complex diazofluorene dimers are distinguished by their symmetric or pseudosymmetric architecture, wherein two halves of the molecule are connected by a sterically hindered C–C bond and adorned with 2–4 unique glycosylated sugars (Figure 1). First isolated in 2001, lomaiviticins A and B quickly became notable for their exceptional bioactivities [1], and subsequent efforts led to the discovery of lomaiviticins C–E in 2012 [2] (Figure 1). Recent microED studies have refined the structural understanding of lomaiviticin C, revising the orientation of the cyclohexenone ring and secondary glycoside configuration in lomaiviticins [6]. Biosynthetically, lomaiviticins share a lineage with kinamycins [7,8,9,10,11,12,13,14] (Figure 1), another class of diazofluorene-containing natural products. However, while kinamycins are sourced from terrestrial *Streptomyces* species [7,8,9,10,11], lomaiviticins originate from marine actinomycetes such as *Micromonospora lomaivitiensis* and *Salinispora* species [1,2,15,16]. A distinguishing biosynthetic hallmark of lomaiviticins is the dimerization of the diazofluorene core, which was proposed to be catalyzed by homologs of ActVA-orf4, such as Lom19 in *Salinispora pacifica* [15] and Strop2191 in *Salinispora tropica* [16]. This unique dimerization significantly contributes to their enhanced bioactivity. Functionally, lomaiviticins A and B are remarkable for their ability to induce double-strand DNA breaks through mechanisms involving vinyl radical intermediates, leveraging their dual diazofluorene moieties to coordinate efficient DNA backbone scission [17]. In contrast, the mono-diazofluorene lomaiviticins C–E lack this robust DNA-damaging capability and exhibit comparatively lower cytotoxicity. The rarity of lomaiviticins in nature, coupled with the challenges of their total synthesis, offers unique opportunities for the discovery of new analogs through both natural product exploration and innovative synthetic methodologies.

**Figure 2:**
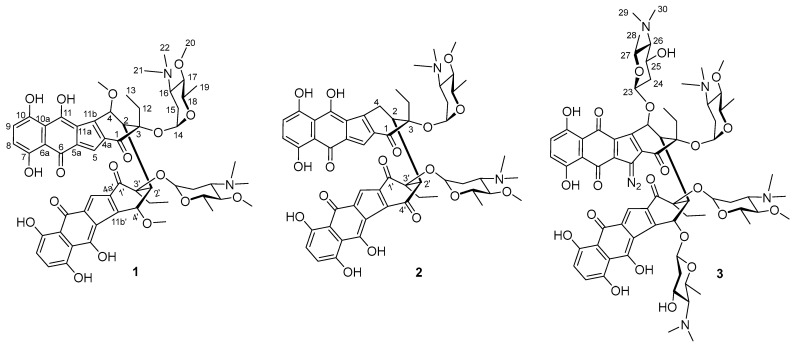
Structures of lomaiviticins F–H (**1**–**3**). Lomaiviticin H (**3**) contains only one diazofluorene moiety, whereas lomaiviticins F and G contain none.

While a range of HRMS-based tools has accelerated the discovery of new natural products, dereplication using HRMS data remains challenging, as accurate mass alone is often insufficient to reliably determine a molecular formula. Recently, we demonstrated the utility of IFS analysis as a powerful approach for determining the exact molecular formulas of natural products [18,19]. IFS holds significant promise for improving the accuracy of dereplication strategies, even when working with crude extracts. The ultrahigh resolving power of FT-ICR-MS enabled the determination of exact molecular formulas of the three intriguing molecules from the crude extract of WMMC-274, leading to the discovery of three new dimeric benzofluorene glycosides, lomaiviticins F–H (**1**–**3**) (Figure 2). Here, we describe the isolation, structure elucidation and biological evaluation for **1**–**3**.

## 2. Results and Discussion

### 2.1. IFS-Based Dereplication

**Figure 3:**
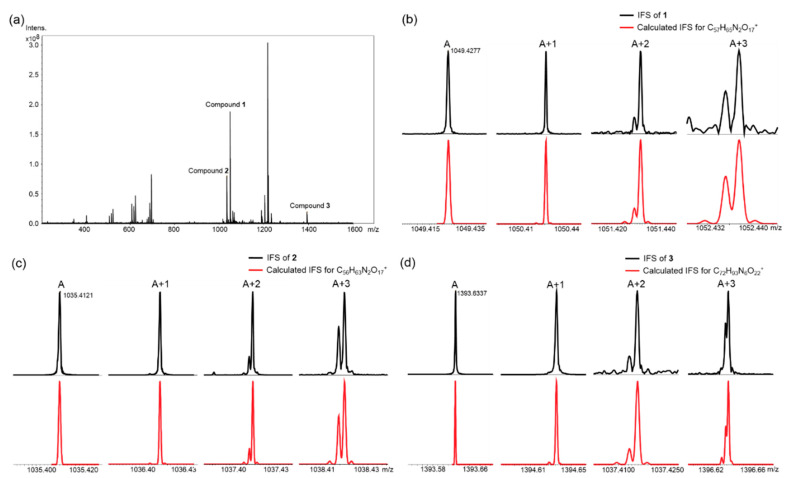
IFS of lomaiviticins F–H (**1**–**3**). (**a**) FT-ICR-MS data of the crude extract sample of strain WMMC-274. (**b**) Comparison of IFS of compound **1** ([M + H − CH_4_O]^+^ with calculated IFS for C_57_H_65_N_2_O_17_^+^. (**c**) Comparison of IFS of compound **2** ([M + H]^+^) with calculated IFS for C_56_H_63_N_2_O_17_^+^. (**d**) Comparison of IFS of compound **3** ([M + H]^+^) with calculated IFS for C_72_H_93_N_6_O_22_^+^.

Although HR-ESI-LCMS provides rapid insight into potentially interesting molecules, most LCMS data do not provide adequate information for dereplication. Dereplication often proceeds by comparing the accurate mass data (error < 5 ppm) with those of known compounds in a given database. Unambiguous assignment of elemental composition facilitates accurate dereplication and is a critical step en route to the structure determination of unknown compounds. Unfortunately, the correct formula cannot be determined solely on the basis of mass accuracy for monoisotopic peaks [20]. IFS, observed by ultrahigh-resolution FT-ICR-MS, is a unique mass spectral signature resulting from different mass defects of isotopic contributions and their natural abundances. In our previous study, we demonstrated the feasibility of this approach to determine the exact molecular formula of forazoline A; the realization that IFS-generated data differed from that originally published inspired our unambiguous structural revision for forazoline A [18]. It is noteworthy that applications of IFS data are generally limited to pure compounds. However, we have also directly applied FT-ICR-MS to analyses of crude extracts, and as seen here, IFS enabled the determination of the exact molecular formulas of **1**–**3** from the crude exact of WMMC-274 (Figure 3). The exact molecular formulae were effectively utilized in dereplication, significantly streamlining our decision-making process to prioritize molecules from WMMC-274 among a vast array of marine actinomycetes. Notably, **1**–**3**, prioritized with the aid of IFS data, proved to be new lomaiviticins on the basis of database queries (Antibase and Scifinder).

### 2.2. Structure Elucidation

The molecular formula of lomaiviticin F (**1**) was established as C_57_H_64_N_2_O_17_ based on FT-ICR-MS data of the crude extract sample of WMMC-274 (Figure 3b), and HR-ESI-MS was carried out on the pure compound to confirm that the isolated vs. crude molecules detected were identical (Figure S7). Analysis of the ^1^H and ^13^C NMR spectra for **1** (Table 1) in CD_3_OD revealed the presence of 31 protons and 29 carbons, which accounted for less than half of the protons and close to half of the carbons in the molecular formula of **1**, suggesting that **1** was likely a symmetric dimer. The NMR spectroscopic features, including 2 aromatic protons (*δ*_H_ 6.74 and 6.58, *J* = 9.1 Hz) and 10 aromatic carbons (*δ*_C_ 186.2, 184.9, 157.0, 156.5, 128.0, 126.0, 125.8, 123.4, 118.1, 117.1), were characteristic of a 5,8-dihydroxy-1,4-naphthoquinone substructure. HMBC correlations from H-4 to C-2, C-3, C-4a and C-11b, from H-2 to C-1, C-3, C-4 and C-11b, together with the HMBC correlations from H_2_-12 to C-2, C-3 and C-13, established the other key substructure of the cyclohexanone ring. HMBC correlations from H-5 to C-4a, C-5a, C-6, C-11, C-11a and C-11b indicated that C-5 was attached to C-4a and C-5a. Similarly to lomaiviticin C, we did not observe an HMBC correlation from H-4 to C-5 using a ^n^*J*_C,H_ threshold of 8 Hz, and a COSY correlation between H-2 and H-4 was observed [6], which completed the orientation of the cyclohexanone substructure with respect to the five membered ring system. We assigned a C-4 linked methoxy group on the basis of the HMBC correlations from H_3_-4-OMe to C-4. The COSY NMR data for **1** showed a series of correlations establishing the sugar moiety H-14–H_3_-19. The placements of the *N*,*N*-dimethylamino and methoxy group at C-16 and C-17 were revealed by HMBC correlations from H_3_-21 and H_3_-22 to C-16, and from H_3_-20 to C-17, respectively. In turn, HMBC correlations from H-14 to C-3 indicated that the sugar moiety was connected to C-3. There were three exchangeable protons attached to three oxygenated aromatic carbons (C-7, C-10 and C-11), which were assigned as 7-OH, 10-OH and 11-OH to complete the monomer structure of **1**. While a molecular formula of C_29_H_34_NO_9_ accounted for the monomer as interpreted by 1D and 2D NMR, obviously, due to the symmetry of compound **1**, the molecular formula C_57_H_64_N_2_O_17_ proposed early on was likely the in-source fragment of the actual molecular formula C_58_H_68_N_2_O_18_. We postulated that the methoxy group was easily lost under MS conditions, causing the observed mass data to be *m*/*z* 32 less than the parent ion; this theory was further confirmed by ESI MS/MS data for compound **1** (Figure S8). Analysis of the MS/MS of **1** revealed mass fragments with differences in *m*/*z* 32 (lost CH_4_O) and *m*/*z* 189 (accounting for one sugar unit) from the parent ion. Ultimately, the two identical monomer units were established as being linked to each other by a C–C bond involving C-2 and C-2′ on the basis of HMBC correlations from H-2 to C-2′.

**Figure 4:**
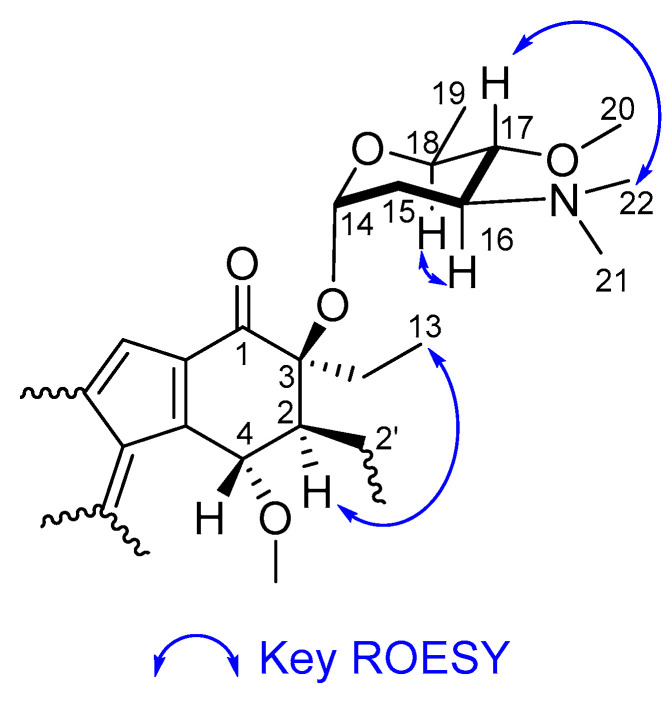
Key ROESY correlations for **1**.

The relative configuration of **1** was determined using a combination of ROESY data (Figure 4) and coupling constants. ROESY correlations from H-2 (2′) to H_3_-13 (13′) indicated their coplanarity within the cyclohexanone ring system. Furthermore, both H-2 and H-4 were observed as singlets in the ^1^H spectrum, consistent with the singlets observed for H-2 (2′) and H-4 (4′) in lomaiviticin C, indicating that H-2 (2′) and H-4 (4′) have the same relative configuration as in lomaiviticin C. Hence, the relative configurations of C-2 (2′), C-3 (3′), and C-4 (4′) were assigned as being identical compared to the known lomaiviticins. In the two-amino-sugar unit attached to C-3 and C-3′, the anomeric proton H-14 (14′) was assigned to be in an α-configuration based on the small coupling constant (*J* = 2.6 Hz) between H-14 (14′) and H_2_-15 (15′). The large coupling constants between H-16 (16′) and H-17 (17′) (*J* = 9.4 Hz), and between H-17 (17′) and H-18 (18′) (*J* = 9.4 Hz) implied that all three protons H-16 (16′), H-17 (17′) and H-18 (18′) were in the axial position, consistent with the ROESY correlations from H-16 (16′) to H-18 (18′) and from H-17 (17′) to the N-CH_3_ groups at C-16 (16′); these data established the sugar residues of 1 as α-4-*O*-methyl-angolosamine. 4-*O*-methyl-angolosamine, containing a dimethylamino group, has also been found in negestatins A and B [21,22], which have the same benzofluorene core. The absolute configuration of 4-*O*-methyl-angolosamine in negestatin A was tentatively assigned as 4-*O*-methyl-l-angolosamine given the presence of a 3,5-epimerase in the biosynthetic pathway for negestatin A [21]. The absolute configuration of α-4-*O*-methyl-angolosamine in **1** was also determined as l on the basis of optical rotation data for acid hydrolysates of **1** [2,23]. The absolute configuration of the aglycone core of **1** was deduced to be the same as (−)-lomaiviticins A–E on the basis of the large negative optical rotation we observed for **1**, while the optical rotation values for (−)-lomaiviticins A–E have all been reported as negative [1,2].

IFS (Figure 3c) of lomaiviticin G (**2**) established its molecular formula as C_56_H_62_N_2_O_17_, and the pure compound was further confirmed by HR-ESI-MS (Figure S20). The ^1^H and ^13^C NMR data of **2** (Table 2) showed many similarities to those of **1**. However, 56 protons were observed for compound **2** in the ^1^H NMR spectrum, especially the presence of six aromatic protons, suggesting that the two putative monomer units in **2** were not identical. Comparisons of the NMR data for **2** with those of **1** revealed the presence of 5,8-dihydroxy-1,4-naphthoquinone and cyclopentadiene substructures, along with the same sugar moieties found in **1** (4-*O*-methyl-angolosamine). However, significantly different structural features were found for the cyclohexanone ring systems in **2**. The ^13^C-^13^C COSY of ^13^C-labeled lomaiviticin G (Table S19) generated by fermentation using uniformly labeled ^13^C glucose quickly established the carbon–carbon connectivity of the remaining substructures. Finally, HMBC correlations from H-2 to C-2′ again showcased the importance of C-2 and C-2′ in constituting the dimer-forming C–C linkage. Meanwhile, the ^13^C-^13^C COSY correlations between C-2 and C-2′ further supported that the two units are connected through the C2–C2′ bond. ROESY correlations between H-2 (2′) and H_3_-13 (13′) confirmed their coplanarity within the cyclohexanone ring system, consistent with the corresponding correlations observed in compound **1**. Furthermore, a comparative analysis of the ^1^H and ^13^C NMR NMR data revealed that the configurations of the α-4-*O*-methyl-angolosamine residues in compound **2** matched those in compound **1**. From a biosynthetic perspective, the absolute configuration of compound **2** around the cyclohexanone ring and the sugar moieties was envisioned to be identical to that found in **1**; this was confirmed by the negative optical rotation value generated with **2**, as had also been the case with compound **1**.

IFS (Figure 3d) of lomaiviticin H (**3**) indicated the molecular formula of **3** as C_72_H_92_N_6_O_22_. Although compound **3** (Table 3) bore some structural similarity to **1** in terms of the ^1^H and ^13^C NMR data, it became apparent early on that **3**, unlike **1**, was not a symmetric dimer. NMR data comparisons revealed that the two methoxy groups attached to C-4 and C-4′ in **1** were each replaced by amino sugar moieties in compound **3**. In addition, COSY data made the presence of the spin systems H-23–H_3_-28 (H-23′–H_3_-28′) clear. Accordingly, *N*,*N*-dimethylamino groups were located at C-26 (26′) as indicated by clear HMBC correlations from H_3_-29 (29′) and H_3_-30 (30′) to C-26 (26′). Interestingly, although the four amino sugar moieties accounted for four nitrogen atoms, there remained two nitrogen atoms unaccounted for. The shielded chemical shift for C-5 (*δ*_C_ 77.9), combined with the clear presence of only one cyclopentadienyl proton (H-5′), suggested that lomaiviticin H contained a diazo group at C-5; comparisons with spectroscopic data for previously reported lomaiviticins A–E supported the assignment of a diazo moiety at C-5 of **3**.

The ^13^C NMR chemical shifts of 4-*O*-methyl-angolosamine in **3** were almost identical to those of **1** and **2**, suggesting that these sugar units had the same α-l configuration as those in **1**. The relative configuration of the other sugar units attached to C-4 and C-4′ were determined by ROESY data and coupling constants. The large coupling constant (10 Hz) observed for H-23 (H-23′), the large coupling constant (10 Hz) between H-25 and H-26, and the large coupling constant (10 Hz) between H-26 and H-27 indicated that protons H-23, H-25, H-26 and H-27 were all in axial positions, allowing us to confidently assign the remaining sugar moieties attached to C-4 and C-4′ of **3** as β-*N*,*N*-dimethyl-pyrrolosamine. The absolute configuration of β-*N*,*N*-dimethyl-pyrrolosamine was assigned as l based upon optical rotation analyses of the TFA hydrolysis product, which shared the same stereochemistry as pyrrolosamine in lomaiviticins A–E [1,2]. The aglycone and 4-*O*-methyl-angolosamine moieties of compound **3** were found to have the same relative configuration as those of compound **1**, based on the analysis of ROESY correlations and ^1^H-^1^H coupling constants, followed by circular dichroism (CD) spectral (Figure S30) and optical rotation analysis. Consequently, compound **3** was deduced to be (−)-lomaiviticin H.

### 2.3. Biological Activity

Lomaiviticins are potent antiproliferative and antimicrobial agents, and it has been demonstrated that the dimeric diazofluorene structure is critical to bioactivity [17]. Lomaiviticin A showed cytotoxicity with IC_50_ values ranging from 0.01 to 98 ng/mL (LNCaP: 2 nM; K562: 11 nM; HCT-116: 2 nM; HeLa: 7 nM) [1,2], and induces double-strand breaks (DSBs) in DNA by a mode of association involving the penetration of both diazofluorene residues into the duplex. The related analogs lomaiviticins C–E, which only contain one or no diazo functional group, do not induce DNA DSBs, implying that DSBs result from two proximal DNA backbone cleaving events, each of which is triggered by diazofluorene-dependent radical chemistry. Predicated on this knowledge, we hypothesized that lomaiviticins F–H, lacking the two diazo functional groups, would be much less toxic against cancer cell lines than lomaiviticin A. Given this hypothesis, we evaluated the cytotoxicity of **1**–**3** against seven cell lines (Table 4). As predicted, the IC_50_ values of lomaiviticins F–H proved significantly higher than those previously noted for lomaiviticin A, thus supporting the notion that the dual diazofluorene moieties in lomaiviticins A and B are vital to cytotoxicity.

Although lomaiviticins F–H exhibit structural differences, their cytotoxic activities remain similarly weak, indicating that these modifications do not lead to a substantial enhancement in biological activity. Notably, lomaiviticin H (**3**), which retains a single diazo functional group, does not demonstrate any significant increase in potency over the other two analogs, further emphasizing that both diazofluorene groups are required to maintain potent cytotoxicity. The drastic reduction in activity observed for lomaiviticins F–H highlights the functional importance of these structural features and suggests that their loss significantly diminishes the ability to engage DNA or trigger cytotoxic mechanisms.

## 3. Conclusions

We employed IFS to facilitate the confirmation of new molecules directly from crude/semi-crude extracts, representing a streamlined approach for rapid and accurate dereplication that can also be linked to bioactivity. Lomaiviticins F–H are structurally most related to the previously known lomaiviticins A–E, and nenestatin B [22] (Figure 1). Specifically, lomaiviticins F–H contain 4-*O*-methyl-L-angolosamine moieties, attached to C-3 and C-3′, which is the same aminosugar unit present in nenestatin B, whereas lomaiviticins A–E bear an oleandrose unit at these positions. Additionally, the location of the bridging bonds between the monomers in nenestatin B and lomaiviticina A–E are distinct. Nenestatin B is characterized as a C2–C1′ asymmetric heterodimer, while lomaiviticins A–E are C2–C2′ dimers. Interestingly, lomaiviticins F–H share the same C-2/C2′ conjoining bond between the monomeric units as lomaiviticins A–E. The discovery of lomaiviticins F–H enriches the chemical diversity of the lomaiviticin family, indicates a close biosynthetic relationship between these two classes lomaiviticins and nenestatins, and provides further insight into the dimerization mechanisms for both families. Finally, cytotoxicity data for lomaiviticins F–H confirm the importance of the diazofluorene moiety in lomaiviticins expressing meaningful anticancer activities; the presence of two diazofluorene moieties appears to be essential for lomaiviticins to exhibit cytotoxicity, supporting the induction of DNA double-strand breaks as a primary mechanism of action for this class of natural products.

## 4. Materials and Methods

### 4.1. General Experimental Procedures

Optical rotations were measured on a Perkin–Elmer 241 Polarimeter (Shelton, CT, USA). UV spectra were recorded on an Aminco/OLIS UV-Vis Spectrophotometer (Bogart, GA, USA). CD spectra were recorded using an AVIV Model 420 Circular Dichroism Spectrometer (Lakewood, NJ, USA). IR spectra were measured with a Bruker Equinox 55/S FT–IR Spectrophotometer (Santa Barbara, CA, USA). Both 1D and 2D NMR spectra were obtained using a Bruker Avance 500 MHz spectrometer with ^1^H{^13^C/^15^N} cryoprobe and 500 MHz spectrometer with ^13^C/^15^N{^1^H} cryoprobe (Billerica, MA, USA); chemical shifts were referenced to the residual solvent peaks (CD_3_OD: *δ*_H_ = 3.31, *δ*_C_ = 49.15). HRMS and MSMS data were acquired with a Bruker MaXis 4G QTOF mass spectrometer (Billerica, MA, USA). FT-ICR-MS and MSMS were performed on a 12-T solariX XR (Bruker, Bremen, Germany) and FT-ICR mass spectrometer equipped with a nano-electrospray ionization source. RP HPLC was performed using a Shimadzu Prominence HPLC system and a Phenomenex Luna C_18_ column (250 × 10 mm, 5 µm) (Torrance, CA, USA).

### 4.2. Biological Material

WMMC-274 was isolated from sponge *Suberites* sp. which were collected on August 7, 2013, near Stan Blum State Park boat launch (27°28′45.7″ N, 80°18′42.8″ W) in Florida, USA. The sponge specimen was taxonomically identified by Mary Kay Harper (University of Utah, Salt Lake City, UT, USA). A voucher specimen is housed at the University of Wisconsin-Madison. For cultivation, sponge samples (1 cm^3^) were ground in 500 μL sterile seawater and then diluted by the addition of 500 μL sterile seawater. Subsequently, 400 μL of diluted sponge sample was added to 200 μL of sterile seawater and 100 μL was plated using a sterile L-shaped spreader. Dilutions were separately plated on six media supplemented with artificial seawater: ISP2, R2A, ISP3, Gauze 1, HV, and Bonito [24,25,26,27,28]. Each medium was supplemented with 50 μg/mL cycloheximide, 25 μg/mL nystatin, and 25 μg/mL nalidixic acid. HV medium was additionally supplemented with 25 μg/mL gentamicin. The plates were incubated at 28 °C and colonies were isolated over the course of two months. Strain WMMC-274 was purified from an HV medium isolation plate [24].

Sequencing: 16S rDNA was sequenced as previously described [29]. WMMC-274 was identified as *Micromonospora* sp., and its genome was deposited to GenBank and assigned the accession number KY015097.1.

Fermentation, extraction, and isolation: Three 10 mL seed cultures (25 × 150 mm tubes) in medium ASW-A (20 g soluble starch, 10 g glucose, 5 g peptone, 5 g yeast extract, 5 g CaCO_3_ per liter of artificial seawater) were inoculated with strain WMMC-274 and agitated at 200 RPM for 14 d at 28 °C. To make artificial seawater, solution I (415.2 g NaCl, 69.54 g Na_2_SO_4_, 11.74 g KCl, 3.40 g NaHCO_3_, 1.7 g KBr, 0.45 g H_3_BO_3_, 0.054 g NaF) and II (187.9 g MgCl_2_•6H_2_O, 22.72 g CaCl_2_•2H_2_O, 0.428 SrCl_2_•6H_2_O) were made up separately, and combined to give a total volume of 20 L. Two-liter flasks (6 × 500 mL) containing ASW-A medium with Diaion HP20 (7% by weight) were inoculated with 10 mL from the culture tube and shaken at 200 rpm at 28 °C for 14 days. Filtered HP20 was washed with distilled H_2_O and extracted with acetone. The acetone extract (8 g) was subjected to a liquid–liquid partitioning using 30% aqueous CH_3_OH and CHCl_3_ (1:1). The CHCl_3_-soluble partition (0.7 g) was fractionated by Sephadex LH20 column chromatography (CHCl_3_:CH_3_OH, 1:1). The fractions containing **1** and **2** were further subjected to RP HPLC (40–70% CH_3_OH-H_2_O with H_2_O over 30 min, 4.0 mg/mL) using a Phenomenex Luna C_18_ column (250 × 10 mm, 5 µm), yielding **1** (10.5 mg, *t*_R_ 17.1 min) and **2** (9 mg, *t*_R_ 19.6 min). The fractions containing **3** were further subjected to RP HPLC (10–80% CH_3_CN-H_2_O with H_2_O containing 0.05% trifluoroacetic acid over 24 min, 4.0 mg/mL) using a Phenomenex Luna C_18_ column (250 × 10 mm, 5 µm), and phosphate buffer (pH = 7) was added to the fraction-collecting tubes before the fraction collection to maintain a stable pH. Collecting tubes that contained compound **3** were then combined and further diluted with water (four times the collection fraction volume) and loaded to a benchtop C_18_ column to remove salts; a column wash with 100% CH_3_CN afforded pure **3** (14.6 mg, *t*_R_ 18.8 min).

*Lomaiviticin F (**1**):* red power; [α]^25^_D_ −225 (*c* 0.2, CH_3_OH); UV (CH_3_OH) λ_max_ (log ε) 205 (3.85), 223 (3.87), 325 (4.03), 476 (3.78) nm; IR (ATR) υ_max_ 3347.9, 2943.4, 2831.8, 1705.2, 1643.6, 1618.5, 1567.2, 1410.8, 1207.9, 1094, 1023.4 cm^−1^; ^1^H and ^13^C NMR (See Table 1); HRESIMS *m*/*z* 1049.4296 [M − CH_4_O + H]^+^ (calcd for C_57_H_65_N_2_O_17_^+^, 1049.4278).

*Lomaiviticin G (**2**):* red power; [α]^25^_D_ −354 (*c* 0.2, CH_3_OH); UV (CH_3_OH) λ_max_ (log ε) 205 (3.96), 237 (3.92), 274 (3.94), 326 (4.04), 476 (3.89) nm; IR (ATR) υ_max_ 3372.8, 2941.6, 2833.4, 1619.6, 1566.4, 1485.8, 1414.9, 1333.2, 1215.5, 1107.8, 1021.3 cm^−1^; ^1^H and ^13^C NMR (See Table S1); HRESIMS *m*/*z* 1035.4151 [M + H]^+^ (calcd for C_56_H_63_N_2_O_17_^+^, 1035.4121).

*Lomaiviticin H (**3**):* red power; [α]_25_^D^ −225 (*c* 0.2, CH_3_OH); UV (CH_3_OH) λ_max_ (log ε) 207 (4.06), 226 (3.78), 320 (4.16), 477 (3.86) nm; IR (ATR) υ_max_ 3344.2, 2943.4, 2832.8, 2140.8, 1618.9, 1567.5, 1486.7, 1453.7, 1412.7, 1335.7, 1205.1, 1095.9, 1024.8 cm^−1^; ^1^H and ^13^C NMR (See Table S2); HRESIMS *m*/*z* 1393.6332 [M + H]^+^ (calcd for C_72_H_93_N_6_O_22_^+^, 1393.6337).

Acid hydrolysis of (–)-Lomaiviticin F (**1**) to generate 4-*O*-methyl-l-angolosamine: According to the published procedure [2], a solution of 0.1 N H_2_SO_4_ (320 µL) was added to a stirred solution of (–)-lomaiviticin F (5.0 mg) in dioxane (445 µL) at room temperature (RT); then, the solution was heated at 50 °C for 40 min. After 40 min, the reaction was cooled down to RT, and dioxane was removed under a stream of dry argon. BaCO_3_ (20 mg) was added to the product to neutralize the reaction and the resulting neutralized mixture was filtered using a syringe filter to remove the insoluble salts. The water was removed under reduced pressure and the remaining residue was loaded to a benchtop C_18_ column. The sugar unit was purified by eluting with 100% water initially, grading to 20% CH_3_CN/H_2_O–water in one step. The optical rotation that we observed for the sugar unit was [α]^25^_D_ −15 (c 0.15, H_2_O).

Hydrolysis of (–)-Lomaiviticin G (**3**) to generate *N*,*N*-dimethyl-l-pyrrolosamine TFA salt: According to the published procedure [2], **3** (5.0 mg) was dissolved in CH_3_OH (900 μL), and 100 mL 10% (*v*/*v*) trifluoroacetic acid was added to the stirred solution of **3** (5.0 mg) at RT. The resulting solution was stirred at RT for 3 h. The product mixture was then concentrated to dryness under a stream of dry argon. A 20 mL aliquot of water was added to the mixture, and the water solution was washed 3× with DCM. The water was removed under reduced pressure and the sugar unit was purified by eluting with 100% water initially, grading to 20% CH_3_CN/H_2_O–water in one step to afford *N*,*N*-dimethyl-l-pyrrolosamine as its trifluoroacetate salt (white solid, 0.7 mg). The optical rotation that we observed for the sugar unit was [α]^25^_D_ −10 (c 0.07, H_2_O).

Cytotoxicity testing: Cell viability for cell lines LnCap, K562, NHDF, A549, MCF7, HCT-116, Hela was determined by the Cell Titer-Glo assay (Promega, Madison, WI, USA) according to the manufacturer’s instructions. The cells were seeded into white 384-well plates (BD falcon) and allowed to attach overnight. The cells were then treated with each compound for 72 h prior to evaluation/cell assessments.

## Figures and Tables

**Table 1:** ^1^H and ^13^C NMR data for lomaiviticin F (**1**) (500 MHz for ^1^H, 125 MHz for ^13^C, CD_3_OD).

Position	*δ*_C_, Mult.	*δ*_H_ (*J* in Hz)	^1^H-^1^H COSY	HMBC ^1^	ROESY
1	201.8, C				
2	43.1, CH	3.78, s	4	1, 3, 4, 11b, 12, 2′	4, 12, 13
3	85.6, C				
4	74.6, CH	4.84, s	2	2, 3, 4a, 5a, 11b, 12-OMe	2
4-OMe	59.1, CH_3_	3.51, s		4	
4a	128.9, C				
5	118.3, CH	7.00, s		1, 4a, 5a, 6, 11, 11a, 11b	
5a	123.4, C				
6	184.9, C				
6a	118.1, C				
7/10	157.0, C/ 156.5, C				
8	126.0, CH	6.74, d (9.1)	9	7, 9, 10, 6a	
9	125.8, CH	6.58, d (9.1)	8	7, 8, 10, 10a	
10a	117.1, C				
11	186.2, C				
11a	128.0, C				
11b	137.0, C				
12	30.9, CH_2_	a: 2.25, m; b: 2.06, m	13	2, 3, 13	2
13	10.0, CH_3_	1.11, t (6.9)	12	3, 12	2, 16, 18
14	94.4, CH	6.11, d (2.6)	15	3, 16, 18	12b, 13
15	31.0, CH_2_	a: 2.45, m; b: 1.82, m	14, 16	14, 16, 17	
16	62.6, CH	3.33, m	15, 17	15, 17, 21, 22	18
17	84.5, CH	2.98, t (9.4)	16, 18	16, 18, 19, 20	15b, 19, 20, 21, 22
18	69.8, CH	4.10, dt (6.2, 9.4)	17, 19	16, 17, 19	16
19	19.6, CH_3_	1.33 d (6.2)	18	17, 18	17, 20
20	60.4, CH_3_	3.56, s		17	15a, 17, 19
21	41.2, CH_3_	2.44, s		16, 22	16, 17, 20
22	41.2, CH_3_	2.44, s		16, 21	16, 17, 20

^1^ HMBC correlations are from proton(s) to the indicated carbon.

**Table 2:** ^1^H, ^13^C and 2D NMR data for **2** (500 MHz for ^1^H, 125 MHz for ^13^C, CD_3_OD).

Position	*δ*_C_, Mult.	*δ*_H_ (*J* in Hz)	^1^H-^1^H COSY	HMBC ^1^	ROESY	^13^C-^13^C COSY
1	200.9, C					3, 4a
2	44.4, CH	3.51, m	4, 2′		13	4, 2′
3	84.6, C					2, 12
4	25.7, CH_2_	a: 3.36, m; b: 2.80, m	2	3, 11b, 2′		2, 11b
4a	129.4, C					1
5	120.1, CH	6.75, s		4a, 5a, 11a, 11b		
5a	121.0, C					6
6	184.8, C					5a, 6a
6a	118.1, C					6, 7
7/10	157.2, C/ 157.0, C					
8	126.3, CH	6.83, d (9.1)	9	7, 10, 6a		7
9	125.7, CH	6.72, d (9.1)	8	7, 10, 10a		10
10a	117.4, C					10
11	185.1, C					11a
11a	127.3, C					11
11b	142.7, C					1
12	27.4, CH_2_	1.98, m; 1.81, m	13			13
13	9.8, CH_3_	1.14, t (7.3)	12	3, 12	1b, 2, 14	12
14	94.1, CH	6.00, br s	15	3, 16, 18	13	15
15	31.6, CH_2_	a: 2.69, m; b: 1.79, m	14, 16			14, 16
16	62.7, CH	3.44, m	15, 17		18	
17	83.6, CH	3.05, t (10.2)	16, 18	16, 18, 19, 20	15b, 19, 21, 22	18
18	69.9, CH	4.04, dt (6.0, 10.2)	17, 19	17	16	17, 19
19	19.4, CH_3_	1.30 d (6.0)	18	17, 18	17, 20	18
20	59.8, CH_3_	3.52, s		17	19	
21	40.9, CH_3_	2.51, s		16, 22	17, 20	
22	40.9, CH_3_	2.51, s		16, 21	17, 20	
1′	200.9, C					3′, 4a′
2′	59.0, CH	4.11, d (2.4)		1′, 3′, 4′, 11b′	13′	2, 4′
3′	85.0, C					2′, 12′
4′	197.0, C					2′, 11b′
4a′	132.7, C					1′
5′	118.2, CH	7.00, s		4′, 4a′, 5a′, 11b′		
5a′	122.3, C					6′
6′	185.4, C					5a′
6a′	117.5, C					7′
7′/10′	158.1, C/ 157.3, C					
8′	127.8, CH	6.95, d (9.1)		7′, 10′, 6a′		7′
9′	127.4, CH	6.90, d (9.1)		7′, 10′, 10a′		10′
10a′	115.7, C					10′, 11′
11′	186.9, C					10a′, 11a′
11a′	131.5, C					11′
11b′	131.0, C					4′
12′	30.3, CH_2_	a: 2.00, m; b: 1.79, m	13′	2′, 3′, 13′		3′, 13′
13′	9.67, CH_3_	1.03, t (7.3)	12′	3′, 12′	2′, 14′	12′
14′	94.0, CH	5.98, br s	15′	3′, 16′, 18′	13′	15′
15′	30.9, CH_2_	2.45, m; 1.82, m	14′, 16′			14′, 16′
16′	62.7, CH	3.26, m	15′, 17′		18′	
17′	83.6, CH	3.01, t (10.5)	16′, 18′	16′, 18′, 19′, 20′	15b′, 19′, 21′, 22′	18′
18′	69.8, CH	4.00, dt (10.5, 6.1)	17′, 19′	17′	16′	17′, 19′
19′	19.3, CH_3_	1.29 d (6.1)	18′	17′, 18′	17′, 20′	18′
20′	59.8, CH_3_	3.52, s		17′	19′	19′
21′	40.5, CH_3_	2.44, s		16′, 22′	17′, 20′	
22′	40.5, CH_3_	2.44, s		16′, 21′	17′, 20′	

^1^ HMBC correlations are from proton(s) to the indicated carbon.

**Table 3:** ^1^H, ^13^C and 2D NMR data for **3** (500 MHz for ^1^H, 125 MHz for ^13^C, CD_3_OD).

Position	*δ*_C_, Mult.	*δ*_H_ (*J* in Hz)	^1^H-^1^H COSY	HMBC ^1^	ROESY
1	201.8, C				
2	43.1, CH	3.78, s	4	1, 3, 4, 11b, 12, 2′	4, 12, 13
3	85.6, C				
4	74.6, CH	4.84, s	2	2, 3, 4a, 5a, 11b, 12-OMe	2, 23
4a	128.9, C				
5	118.3, CH	7.00, s		1, 4a, 5a, 6, 11, 11a, 11b	
5a	123.4, C				
6	184.9, C				
6a	118.1, C				
7/10	157.0, C/ 156.5, C				
8	126.0, CH	6.74, d (9.1)	9	7, 9, 10, 6a	
9	125.8, CH	6.58, d (9.1)	8	7, 8, 10, 10a	
10a	117.1, C				
11	186.2, C				
11a	128.0, C				
11b	137.0, C				
12	30.9, CH_2_	a: 2.25, m; b: 2.06, m	13	2, 3, 13	2
13	10.0, CH_3_	1.11, t (6.9)	12	3, 12	2, 16, 18
14	94.4, CH	6.11, d (2.6)	15	3, 16, 18	12b, 13
15	31.0, CH_2_	a: 2.45, m; b: 1.82, m	14, 16	14, 16, 17	
16	62.6, CH	3.33, m	15, 17	15, 17, 21, 22	18
17	84.5, CH	2.98, t (9.4)	16, 18	16, 18, 19, 20	15b, 19, 20, 21, 22
18	69.8, CH	4.10, dt (6.2, 9.4)	17, 19	16, 17, 19	16
19	19.6, CH_3_	1.33 d (6.2)	18	17, 18	17, 20
20	60.4, CH_3_	3.56, s		17	15a, 17, 19
21	41.2, CH_3_	2.44, s		16, 22	16, 17, 20
22	41.2, CH_3_	2.44, s		16, 21	16, 17, 20
1′	201.8, C				
2′	43.1, CH	3.78, s	4′	1′, 3′, 4′, 11b′, 12′, 2	4′, 12′, 13′
3′	85.6, C				
4′	74.6, CH	4.84, s	2′	2′, 3′, 4a′, 5a′, 11b′, 12′-OMe	2′, 23′
4a′	128.9, C				
5′	118.3, CH	7.00, s		1′, 4a′, 5a′, 6′, 11′, 11b′	
5a′	123.4, C				
6′	184.9, C				
6a′	118.1, C				
7′/10′	157.0, C/ 156.5, C				
8′	126.0, CH	6.74, d (9.1)	9′	7′, 9′, 10′, 6a′	
9′	125.8, CH	6.58, d (9.1)	8′	7′, 8′, 10′, 10a′	
10a′	117.1, C				
11′	186.2, C				
11a′	128.0, C				
11b′	137.0, C				
12′	30.9, CH_2_	2.25, m; 2.06, m	13′	2′, 3′, 13′	2′
13′	10.0, CH_3_	1.11, t (6.9)	12′	3′, 12′	2′, 16′, 18′
14′	94.4, CH	6.11, d (2.6)	15′	3′, 16′, 18′	12b′, 13′
15′	31.0, CH_2_	2.45, m; 1.82, m	14′, 16′	14′, 16′, 17′	
16′	62.6, CH	3.33, m	15′, 17′	15′, 17′, 21′, 22′	18′
17′	84.5, CH	2.98, m	16′, 18′	16′, 18′, 19′, 20′	15b′, 19′, 20′, 21′, 22′
18′	69.8, CH	4.10, m	17′, 19′	16′, 17′, 19′	16′
19′	19.6, CH_3_	1.33 d (6.2)	18′	17′, 18′	17′, 20′
20′	60.4, CH_3_	3.56, s		17′	15a′, 17′, 19′
21′	41.2, CH_3_	2.44, s		16′, 22′	16′, 17′, 20′
22′	41.2, CH_3_	2.44, s		16′, 21′	16′, 17′, 20′

^1^ HMBC correlations are from proton(s) to the indicated carbon.

**Table 4:** IC_50_ values (µM) of lomaiviticins F–H (**1**–**3**) against cancer cell lines.

Cell Lines	1	2	3
LNCaP	>20	>20	>20
K562	>20	5.75	>20
NHDF	>20	>20	>20
A549	9.4	8.34	12
MCF7	5.4	>20	>20
HCT-116	>20	>20	9.79
HeLa	11.1	10.6	13.1

## Data Availability

The original data presented in the study are included in the article/Supplementary Material; further inquiries can be directed to the corresponding author.

## Supplementary Materials

The following supporting information can be downloaded at: https://www.mdpi.com/article/10.3390/md23020065/s1, Figure S1: ^1^H NMR spectrum of lomaiviticin F (**1**; 500 MHz, CD_3_OD), Figure S2: ^13^C NMR spectrum of lomaiviticin F (**1**; 125 MHz, CD_3_OD), Figure S3: gCOSY spectrum of lomaiviticin F (**1**; 500 MHz, CD_3_OD), Figure S4: gHSQC spectrum of lomaiviticin F (**1**; 500 MHz, CD_3_OD), Figure S5: gHMBC spectrum of lomaiviticin F (**1**; 500 MHz, CD_3_OD); Figure S6: ROSEY Spectrum of lomaiviticin F (**1**; 500 MHz, CD_3_OD), Figure S7: Positive ion HRESIMS of lomaiviticin F (**1**), Figure S8: Positive ion ESI-MS/MS spectrum of lomaiviticin F (**1**), Figure S9: ^1^H NMR spectrum of lomaiviticin G (**2**; 500 MHz, CD_3_OD), Figure S10: ^13^C NMR spectrum of lomaiviticin G (**2**; 125 MHz, CD_3_OD), Figure S11: gCOSY spectrum of lomaiviticin G (**2**; 500 MHz, CD_3_OD), Figure S12: gHSQC spectrum of lomaiviticin G (**2**; 500 MHz, CD_3_OD), Figure S13: gHMBC spectrum of lomaiviticin G (**2**; 500 MHz, CD_3_OD), Figure S14: ROSEY spectrum of lomaiviticin G (**2**; 500 MHz, CD_3_OD), Figure S15. ^1^H NMR spectrum of ^13^C labeled lomaiviticin G (**2**; 500 MHz, CD_3_OD), Figure S16: ^13^C NMR spectrum of ^13^C labeled lomaiviticin G (**2**; 125 MHz, CD_3_OD), Figure S17: ^1^H NMR spectrum comparation between ^13^C labeled (top) and unlabeled (bottom) lomaiviticin G (**2**; 500 MHz, CD_3_OD), Figure S18: ^13^C NMR spectrum comparation between ^13^C labeled (top) and unlabeled (bottom) lomaiviticin G (**2**; 125 MHz, CD_3_OD), Figure S19: ^13^C-^13^C COSY spectrum of ^13^C labeled lomaiviticin G (**2**; 125 MHz, CD_3_OD), Figure S20: Positive ion HRESIMS of lomaiviticin G (**2**), Figure S21: Positive ion ESI-MS/MS spectrum of lomaiviticin G (**2**), Figure S22: ^1^H NMR spectrum of lomaiviticin H (**3**; 500 MHz, CD_3_OD), Figure S23: ^13^C NMR spectrum of lomaiviticin H (**3**; 125 MHz, CD_3_OD), Figure S24: gCOSY spectrum of lomaiviticin H (**3**; 500 MHz, CD_3_OD), Figure S25: gHSQC spectrum of lomaiviticin H (**3**; 500 MHz, CD_3_OD), Figure S26: gHMBC spectrum of lomaiviticin H (**3**; 500 MHz, CD_3_OD), Figure S27: ROSEY spectrum of lomaiviticin H (**3**; 500 MHz, CD_3_OD), Figure S28: Positive ion HRESIMS of lomaiviticin H (**3**), Figure S29: Positive ion ESI-MS/MS spectrum of lomaiviticin H (**3**), Figure S30: CD spectra of **1**–**3**.

## Abbreviations

The following abbreviations are used in this manuscript:

IFS	Isotopic Fine Structure
DSB	Double-Strand Breaks
