# Supplementary material for: Isolation, Structure Elucidation and Biological Evaluation of Lomaiviticins F–H, Dimeric Benzofluorene Glycosides from Marine-Derived Micromonospora sp. Bacterium
Source: Mar Drugs. 2025 Feb 3;23(2):65. doi: 10.3390/md23020065 (PMC11857394; doi:10.3390/md23020065)

Isolation, Structure Elucidation and Biological Evaluation of Lomaiviticins F–H, Dimeric Benzofluorene Glycosides from Marine-Derived *Micromonospora* sp. Bacterium

Fan Zhang [1,2,*], Wenhui Wang [1], Doug R. Braun [2], Gene E. Ananiev [3], Weiting Liao [1], Mary Kay Harper [4], Scott R. Rajski [2] and Tim S. Bugni [2]

[1] Department of Pulmonary and Critical Care Medicine, Zhongnan Hospital of Wuhan University,
TaiKang Center for Life and Medical Sciences, School of Pharmaceutical Sciences,
Key Laboratory of Combinatorial Biosynthesis and Drug Discovery, Ministry of Education, Wuhan University, Wuhan 430071, China; 2022203060042@whu.edu.cn (W.W.); weitingl@whu.edu.cn (W.L.)

[2] Pharmaceutical Sciences Division, University of Wisconsin–Madison, Madison, WI 53705, USA; drbraun1@wisc.edu (D.R.B.); scott.rajski@wisc.edu (S.R.R.); tim.bugni@wisc.edu (T.S.B.)

[3] Small Molecule Screening & Synthesis Facility, UW Carbone Cancer Center, Madison, WI 53705, USA;
geananiev@wisc.edu

[4] Department of Medicinal Chemistry, University of Utah, 30 South 2000 East, Salt Lake City, UT 84112, USA; mk.harper@pharm.utah.edu

* Correspondence: fzhang83@whu.edu.cn

Supporting Information Table of Contents

Contents **Page**

1) **Figure S1.** ^{1}H NMR spectrum of lomaiviticin F (**1**; 500 MHz, CD_3OD).................. 3
2) **Figure S2.** ^{13}C NMR spectrum of lomaiviticin F (**1**; 125 MHz, CD_3OD)................. 4
3) **Figure S3.** gCOSY spectrum of lomaiviticin F (**1**; 500 MHz, CD_3OD).................... 5
4) **Figure S4.** gHSQC spectrum of lomaiviticin F (**1**; 500 MHz, CD_3OD).................... 6
5) **Figure S5.** gHMBC spectrum of lomaiviticin F (**1**; 500 MHz, CD_3OD)................... 7
6) **Figure S6.** ROSEY Spectrum of lomaiviticin F (**1**; 500 MHz, CD_3OD) 8
7) **Figure S7.** Positive ion HRESIMS of lomaiviticin F (**1**).. 9
8) **Figure S8.** Positive ion ESI-MS/MS spectrum of lomaiviticin F (**1**)....................... 10
9) **Figure S9.** ^{1}H NMR spectrum of lomaiviticin G (**2**; 500 MHz, CD_3OD)................. 11
10) **Figure S10.** ^{13}C NMR spectrum of lomaiviticin G (**2**; 125 MHz, CD_3OD)................ 12
11) **Figure S11.** gCOSY spectrum of lomaiviticin G (**2**; 500 MHz, CD_3OD).................... 13
12) **Figure S12.** gHSQC spectrum of lomaiviticin G (**2**; 500 MHz, CD_3OD).................... 14
13) **Figure S13.** gHMBC spectrum of lomaiviticin G (**2**; 500 MHz, CD_3OD).................. 15
14) **Figure S14.** ROSEY Spectrum of lomaiviticin G (**2**; 500 MHz, CD_3OD) 16
15) **Figure S15.** ^{1}H NMR spectrum of ^{13}C labeled lomaiviticin G (**2**; 500 MHz, CD_3OD) 17
16) **Figure S16.** ^{13}C NMR spectrum of ^{13}C labeled lomaiviticin G (**2**; 125 MHz, CD_3OD) 18
17) **Figure S17.** ^{1}H NMR spectrum comparation between ^{13}C labeled (top) and unlabeled (bottom) lomaiviticin G (**2**; 500 MHz, CD_3OD) 19
18) **Figure S18.** ^{13}C NMR spectrum comparation between ^{13}C labeled (top) and unlabeled (bottom) lomaiviticin G (**2**; 125 MHz, CD_3OD) 20
19) **Figure S19.** ^{13}C-^{13}C COSY spectrum of ^{13}C labeled lomaiviticin G (**2**; 125 MHz, CD_3OD) 21
20) **Figure S20.** Positive ion HRESIMS of lomaiviticin G (**2**).. 22
21) **Figure S21.** Positive ion ESI-MS/MS spectrum of lomaiviticin G (**2**)....................... 23
22) **Figure S22.** ^{1}H NMR spectrum of lomaiviticin H (**3**; 500 MHz, CD_3OD).................. 24
23) **Figure S23.** ^{13}C NMR spectrum of lomaiviticin H (**3**; 125 MHz, CD_3OD)................. 25
24) **Figure S24.** gCOSY spectrum of lomaiviticin H (**3**; 500 MHz, CD_3OD).................... 26
25) **Figure S25.** gHSQC spectrum of lomaiviticin H (**3**; 500 MHz, CD_3OD).................... 27
26) **Figure S26.** gHMBC spectrum of lomaiviticin H (**3**; 500 MHz, CD_3OD)................... 28
27) **Figure S27.** ROSEY Spectrum of lomaiviticin H (**3**; 500 MHz, CD_3OD) 29
28) **Figure S28.** Positive ion HRESIMS of lomaiviticin H (**3**).. 30
29) **Figure S29.** Positive ion ESI-MS/MS spectrum of lomaiviticin H (**3**)...................... 31
30) **Figure S30.** CD spectra of **1–3**... 32

Figure S1. ^{1}H NMR spectrum of lomaiviticin F (**1**; 500 MHz, CD_3OD)

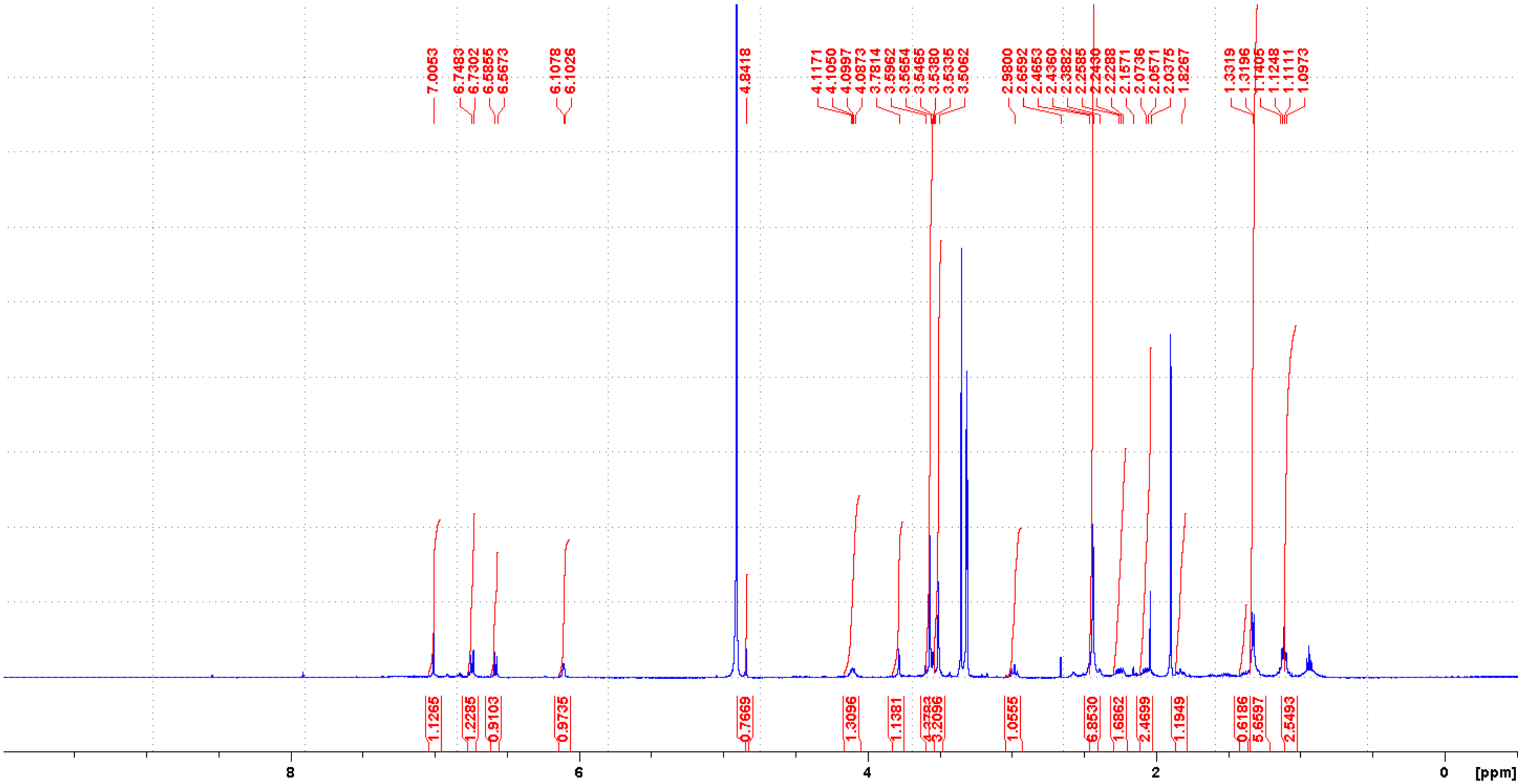

Figure S2. ^{13}C NMR spectrum of lomaiviticin F (**1**; 125 MHz, CD_3OD)

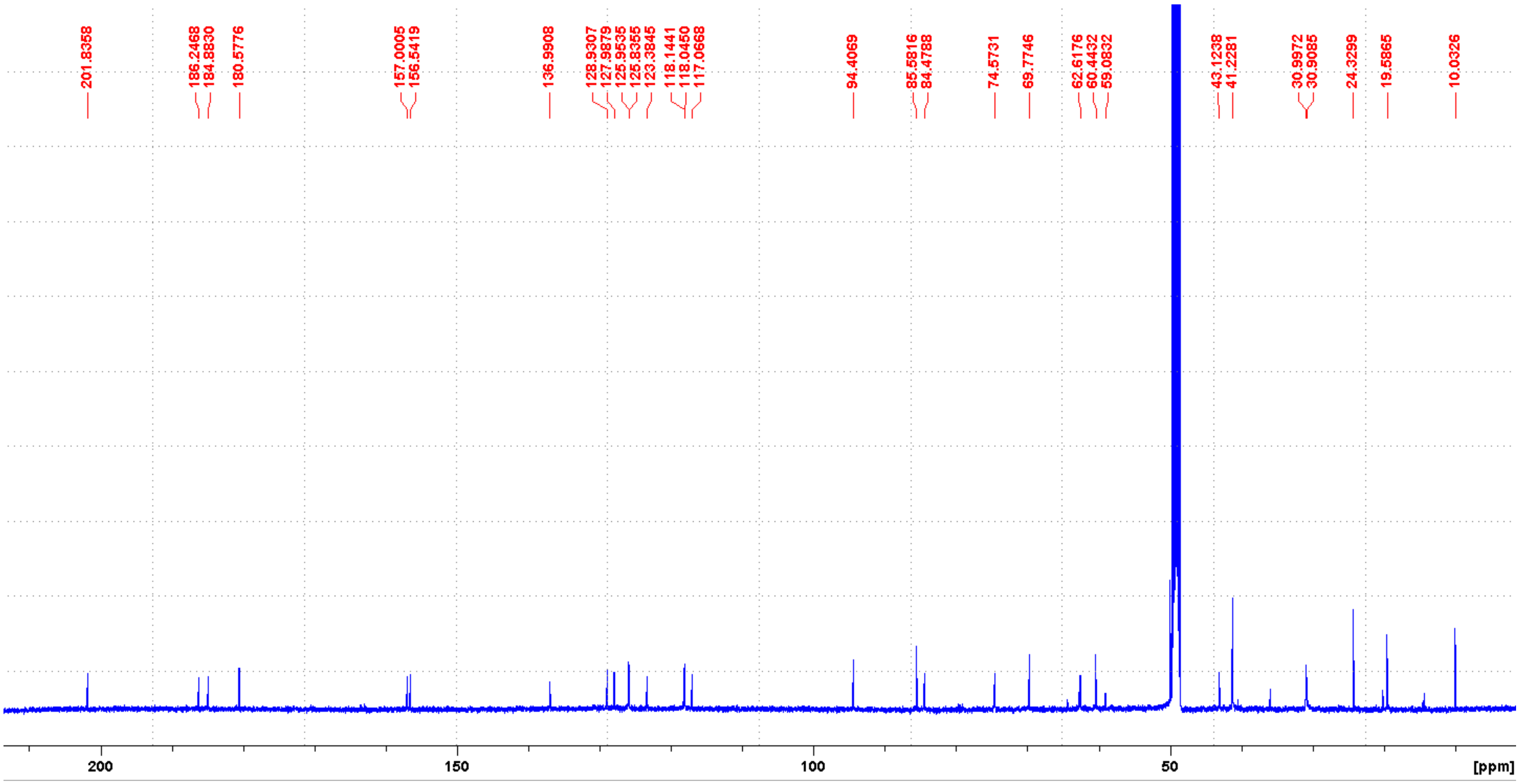

Figure S3. gCOSY spectrum of lomaiviticin F (**1**; 500 MHz, CD_3OD)

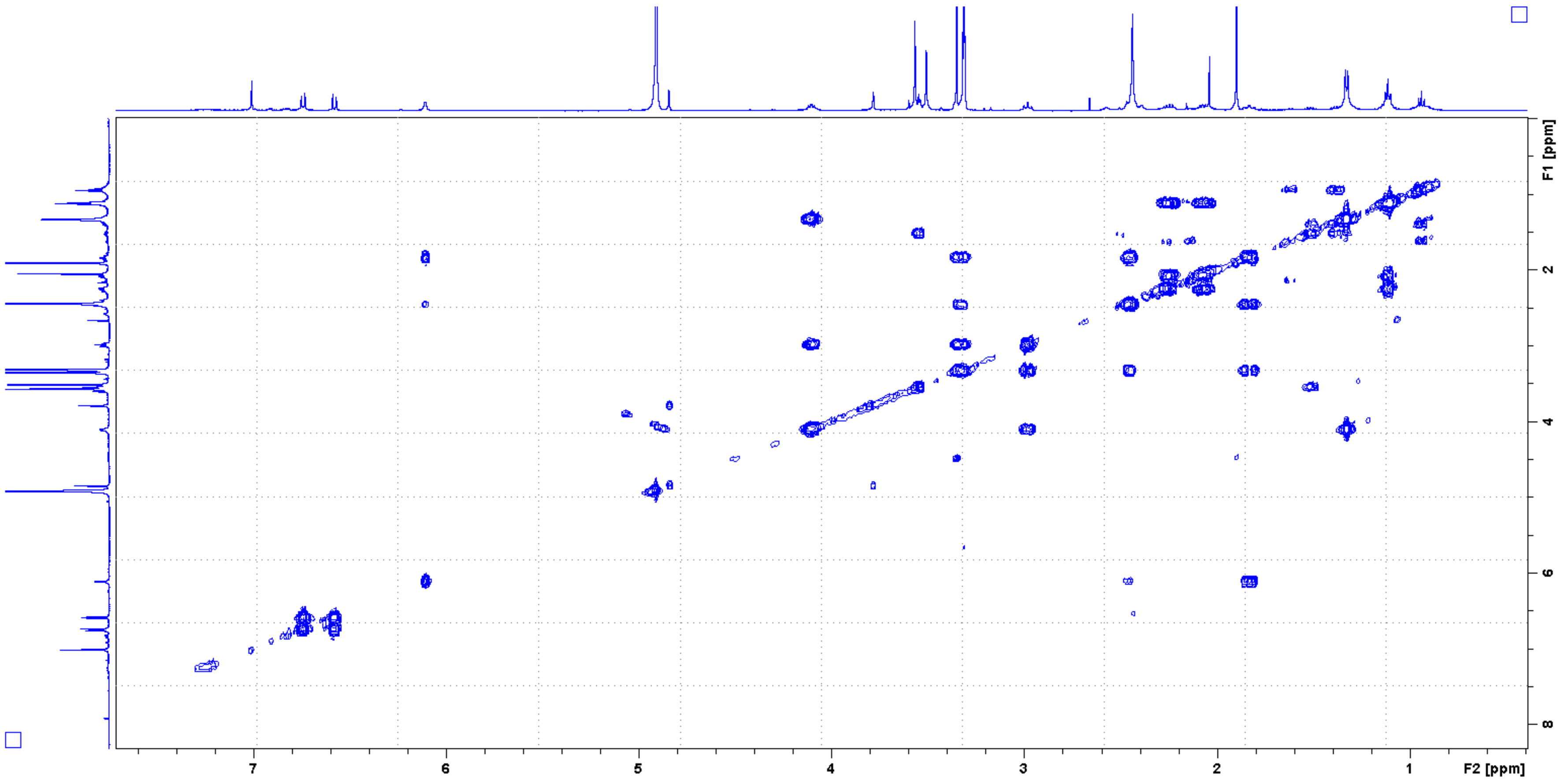

Figure S4. gHSQC spectrum of lomaiviticin F (**1**; 500 MHz, CD_3OD)

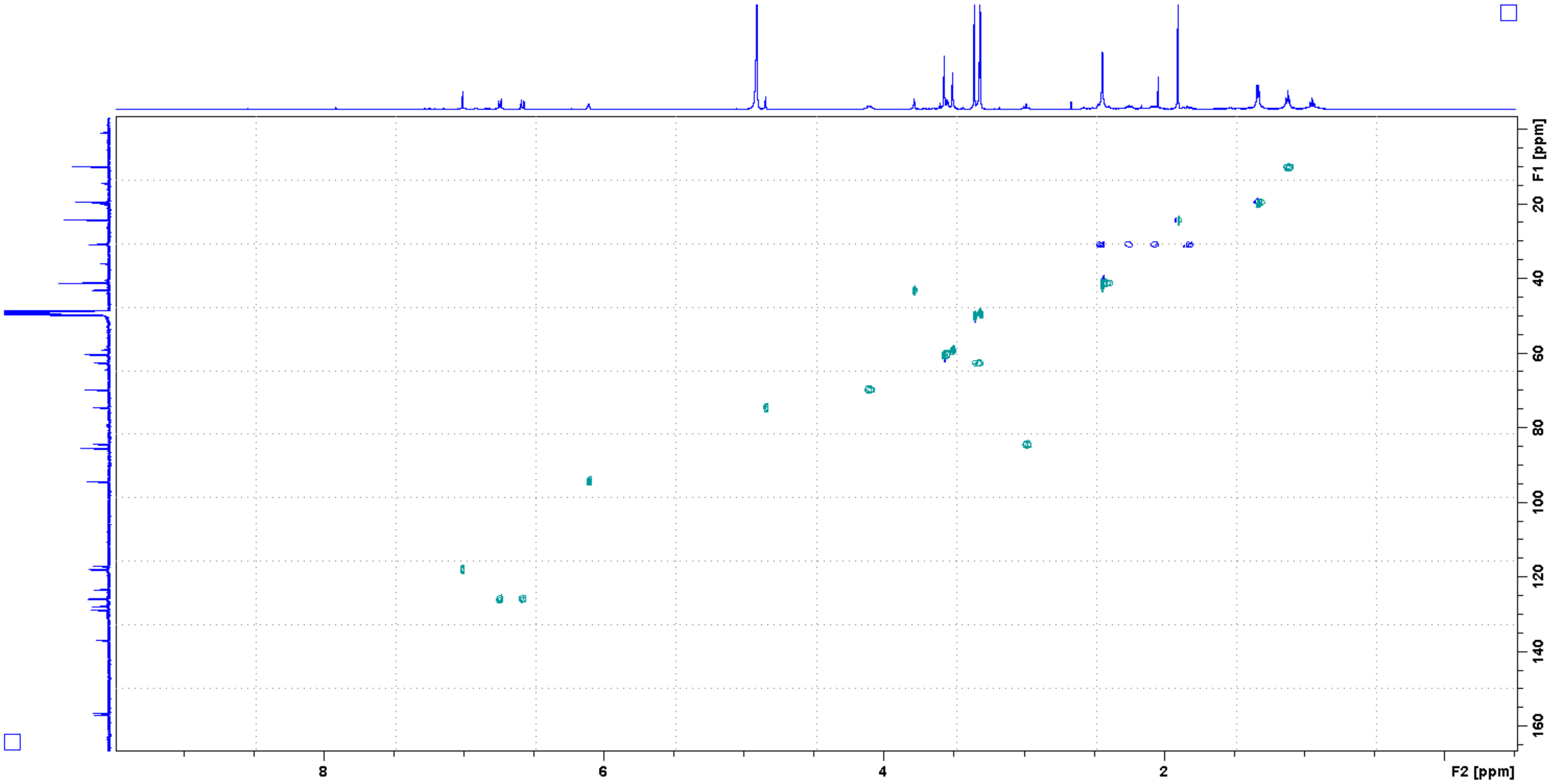

Figure S5. gHMBC spectrum of lomaiviticin F (**1**; 500 MHz, CD_3OD)

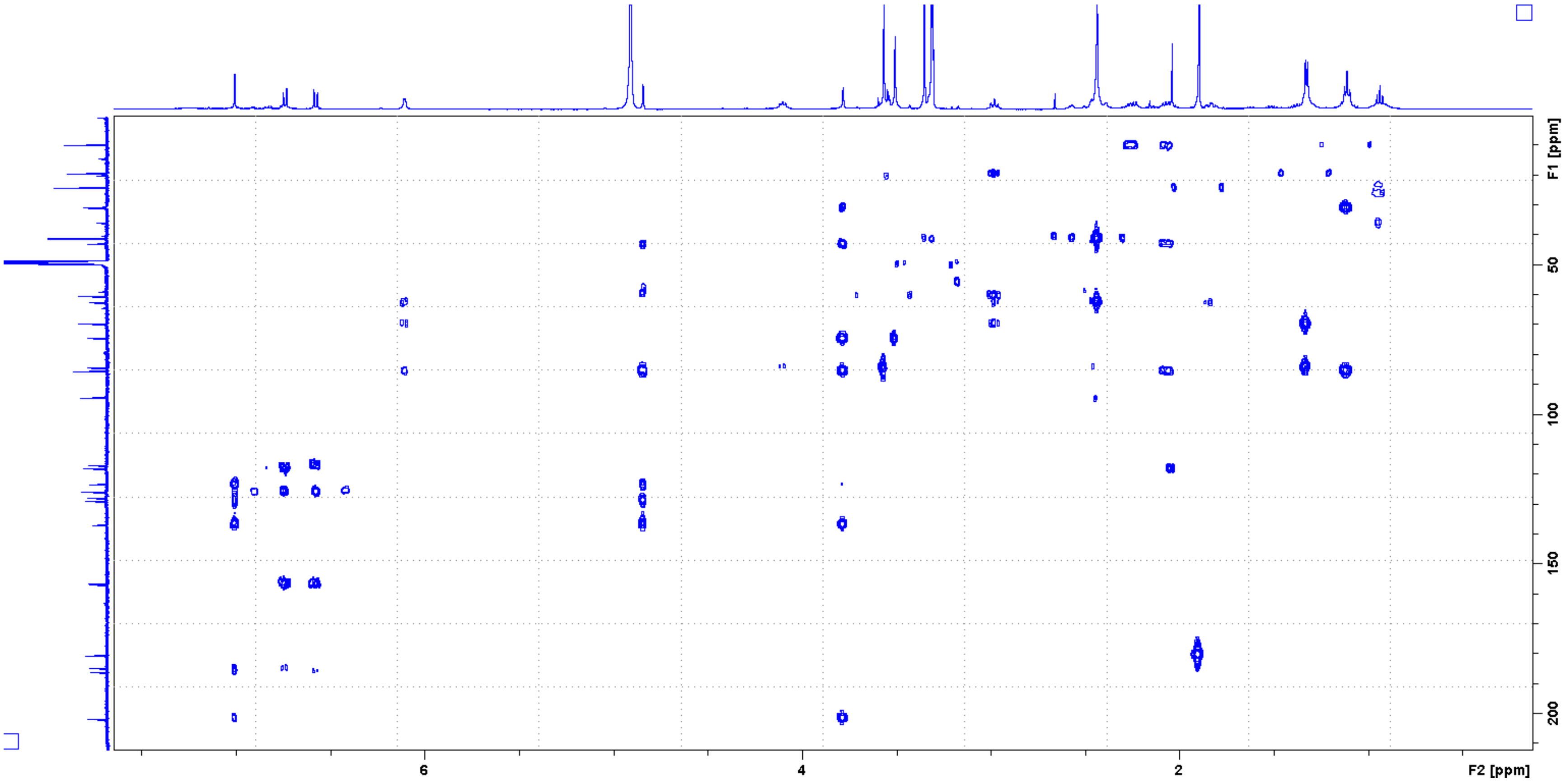

Figure S6. ROSEY Spectrum of lomaiviticin F (**1**; 500 MHz, CD_3OD)

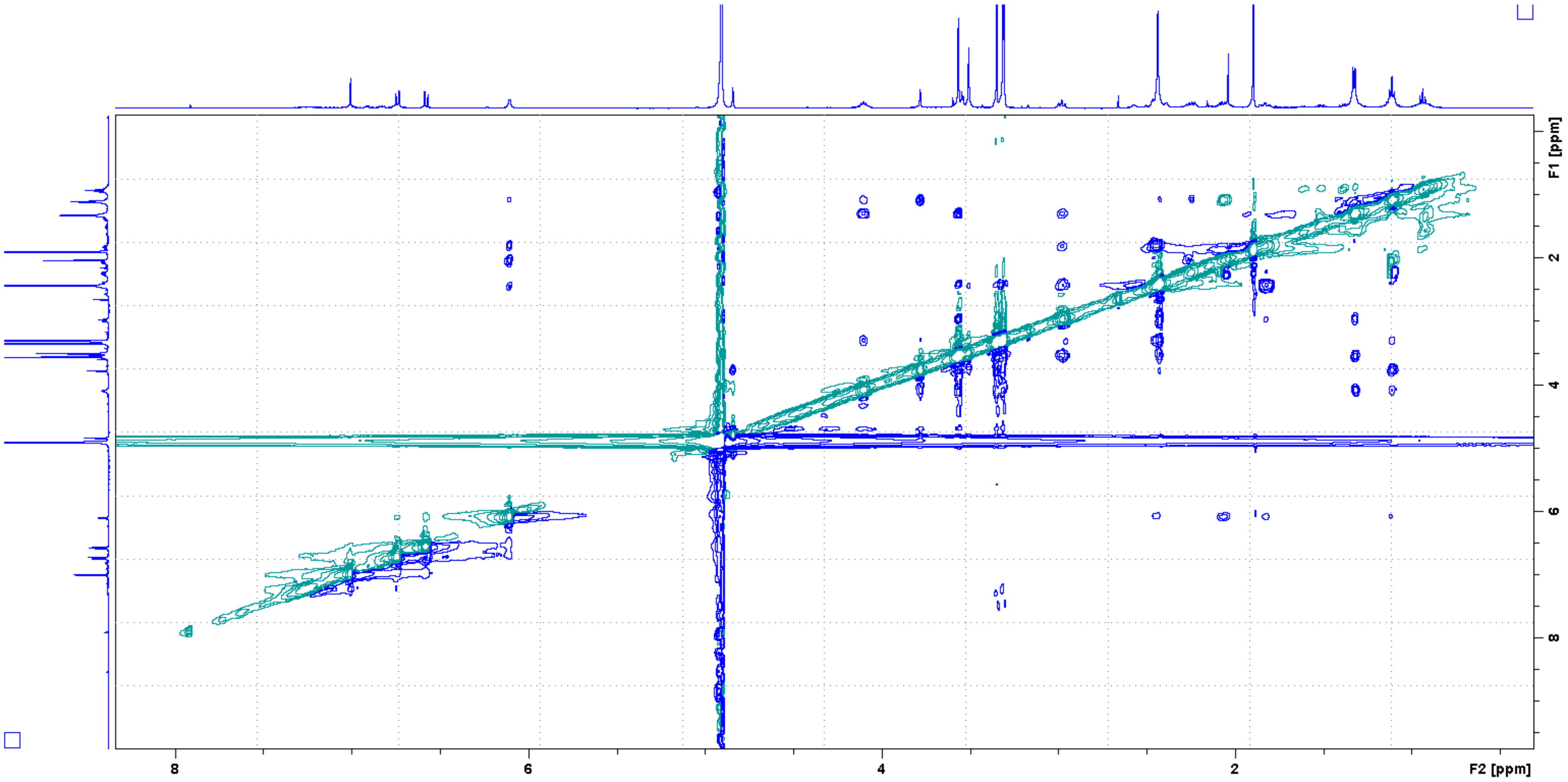

Figure S7. Positive ion HRESIMS of lomaiviticin F (**1**)

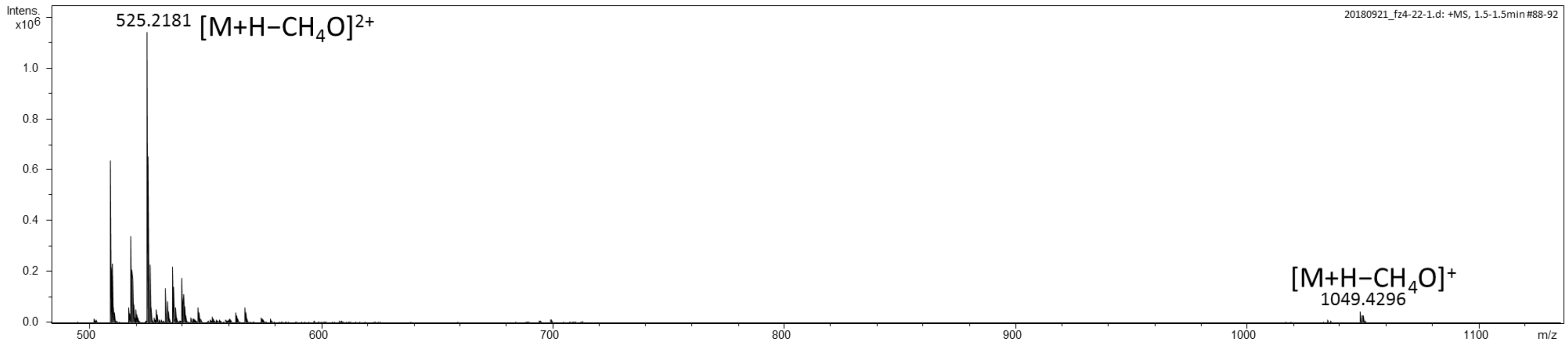

Figure S8. Positive ion ESI-MS/MS spectrum of lomaiviticin F (**1**)

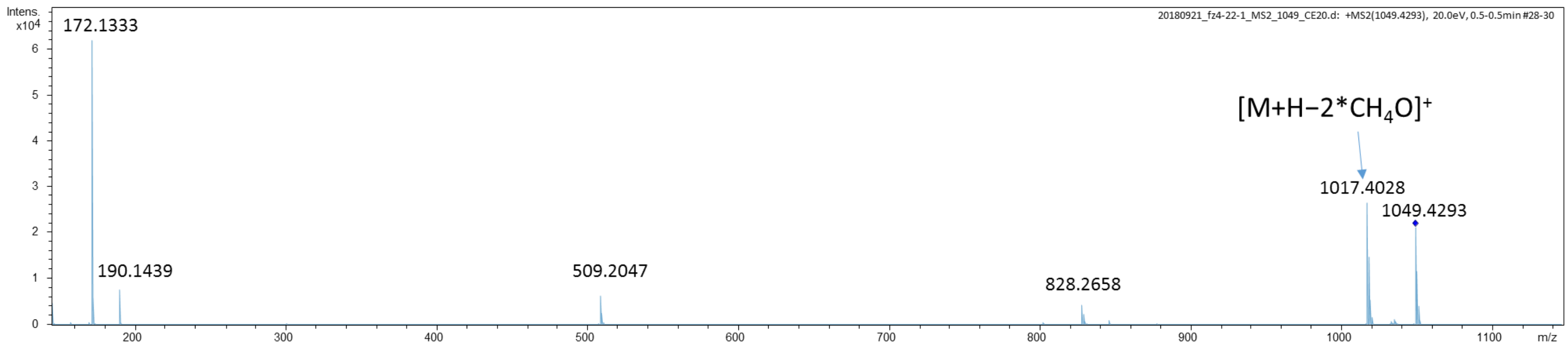

Figure S9. ^{1}H NMR spectrum of lomaiviticin G (**2**; 500 MHz, CD_3OD)

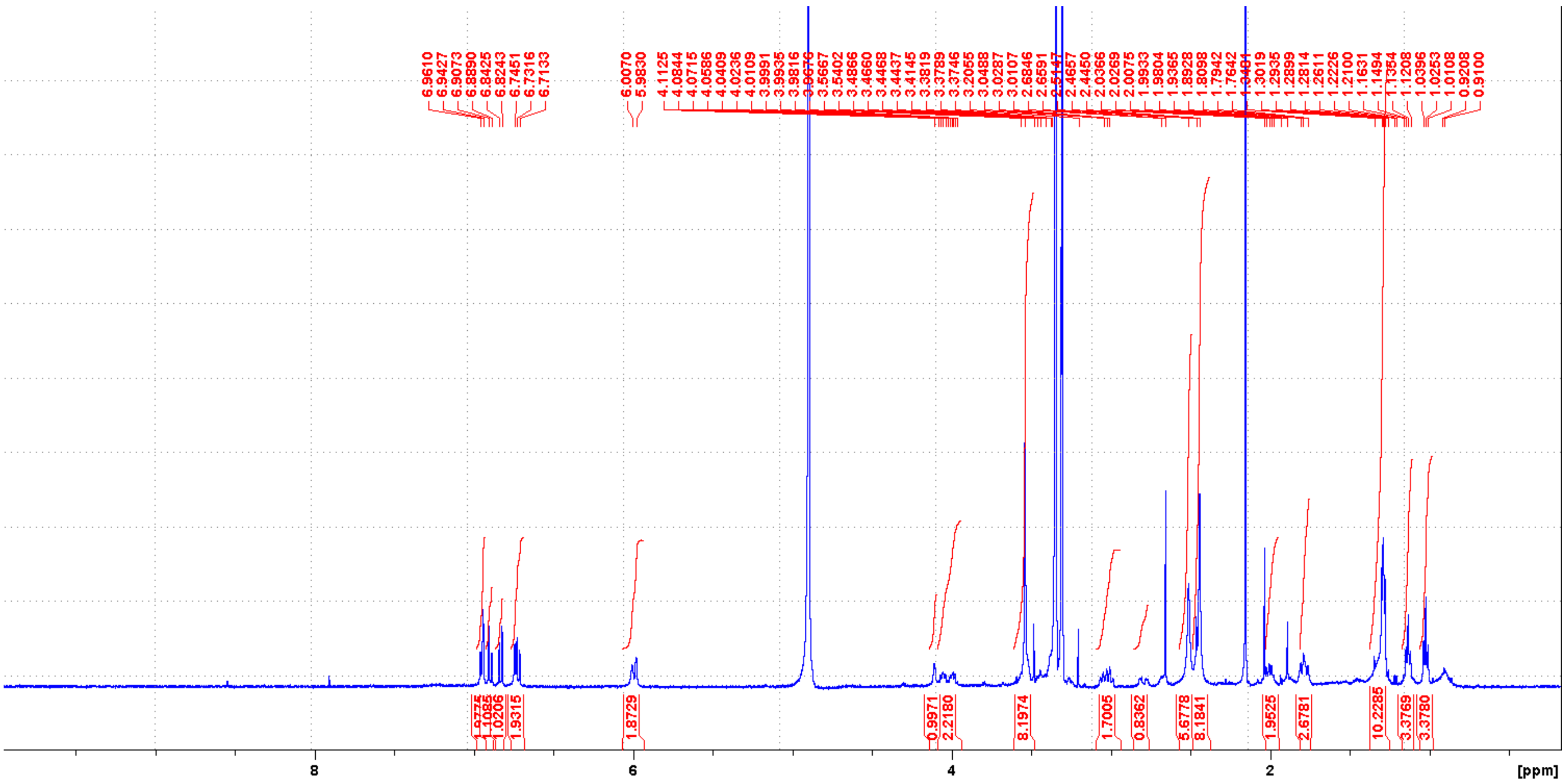

Figure S10. ^{13}C NMR spectrum of lomaiviticin G (**2**; 125 MHz, CD_3OD)

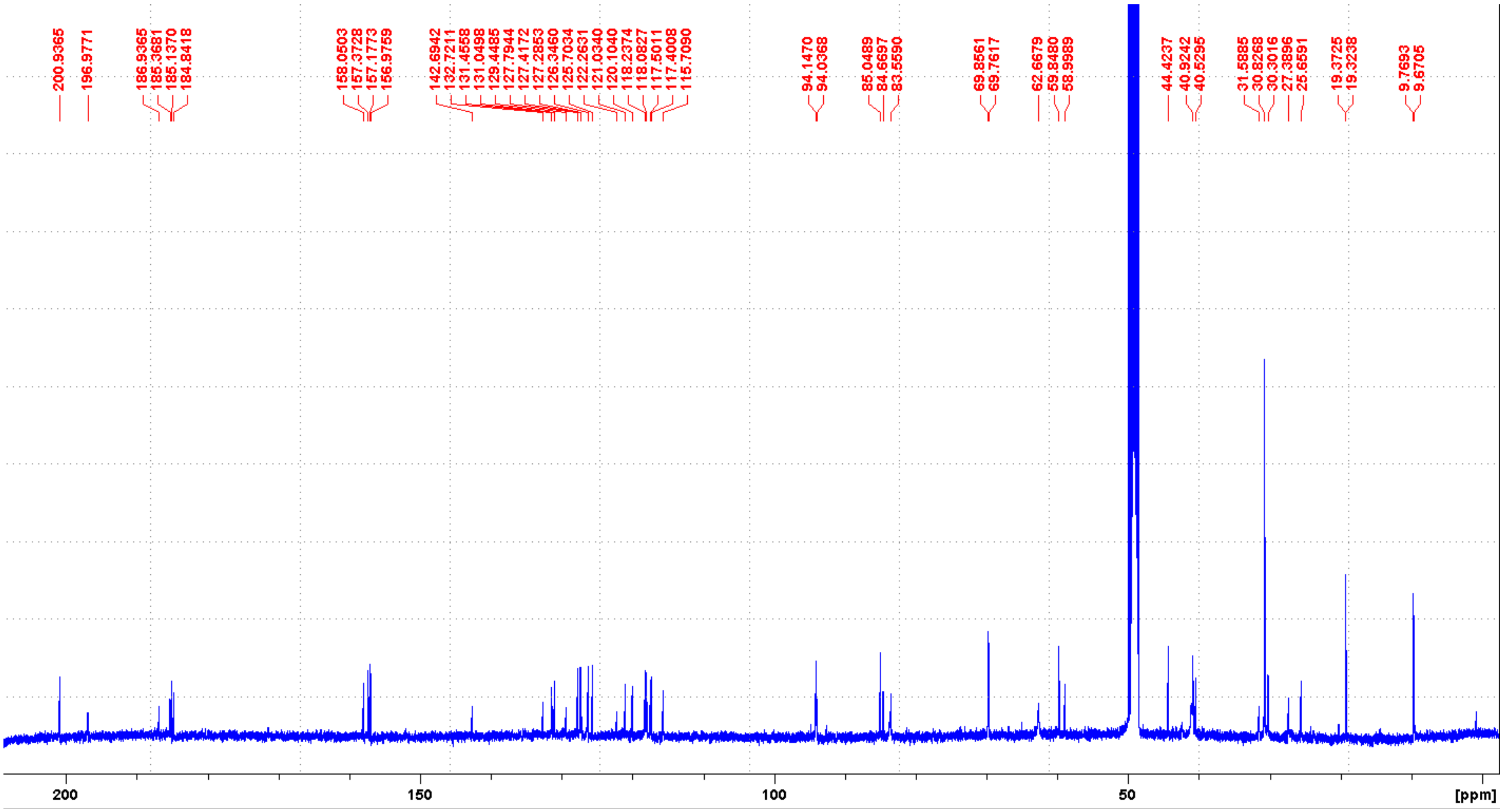

Figure S11. gCOSY spectrum of lomaiviticin G (**2**; 500 MHz, CD_3OD)

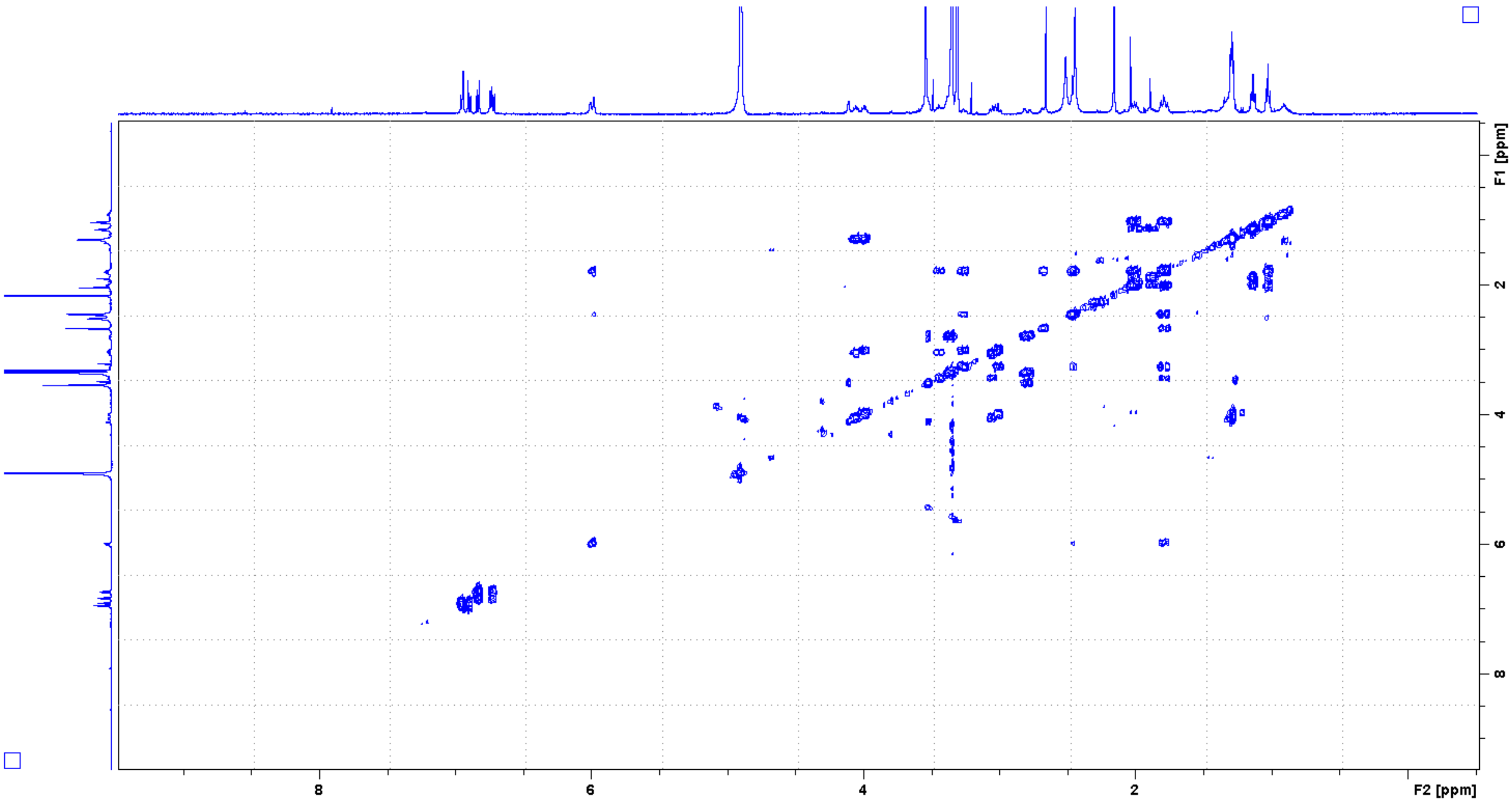

Figure S12. gHSQC spectrum of lomaiviticin G (**2**; 500 MHz, CD_3OD)

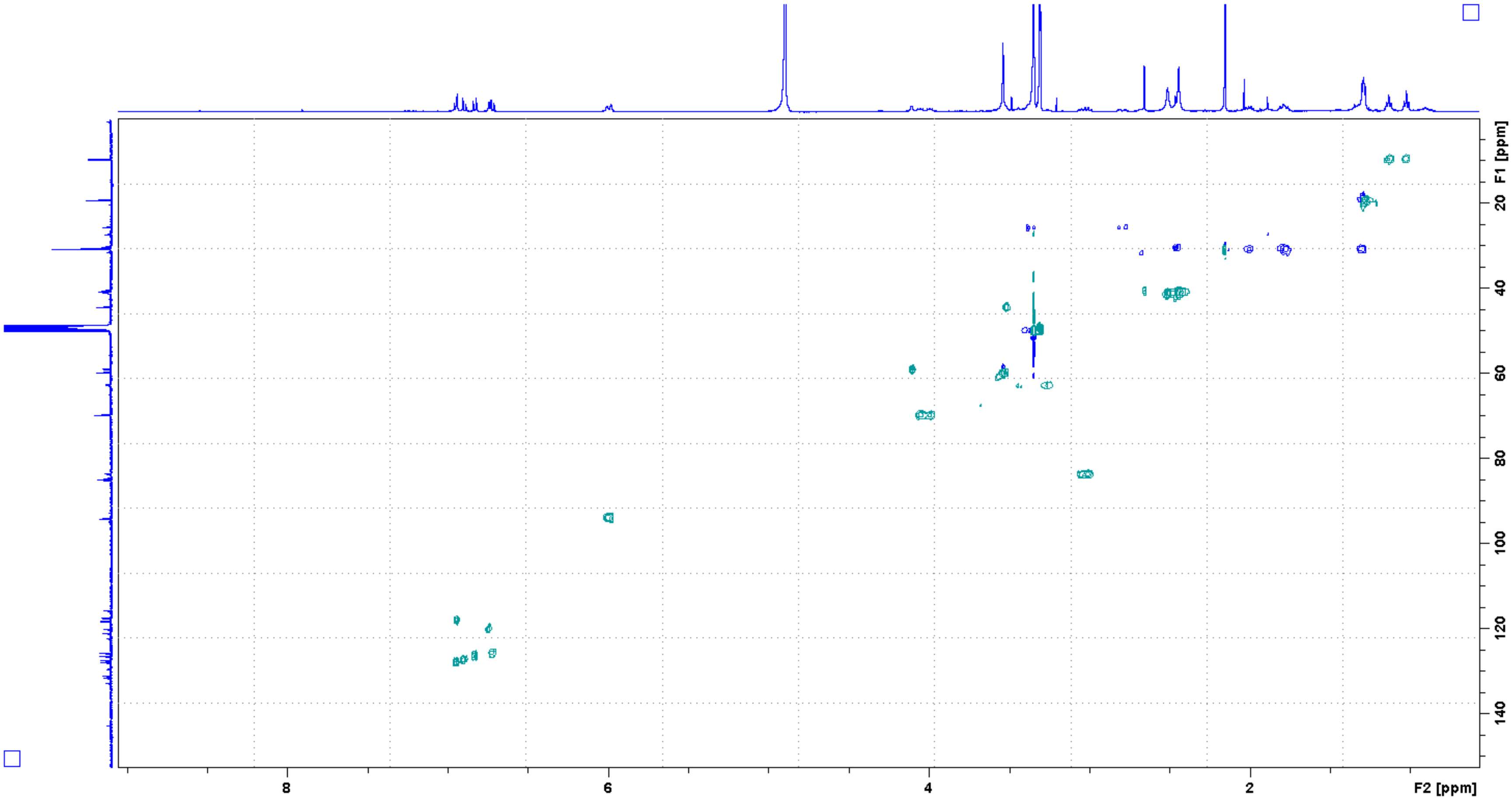

Figure S13. gHMBC spectrum of lomaiviticin G (**2**; 500 MHz, CD_3OD)

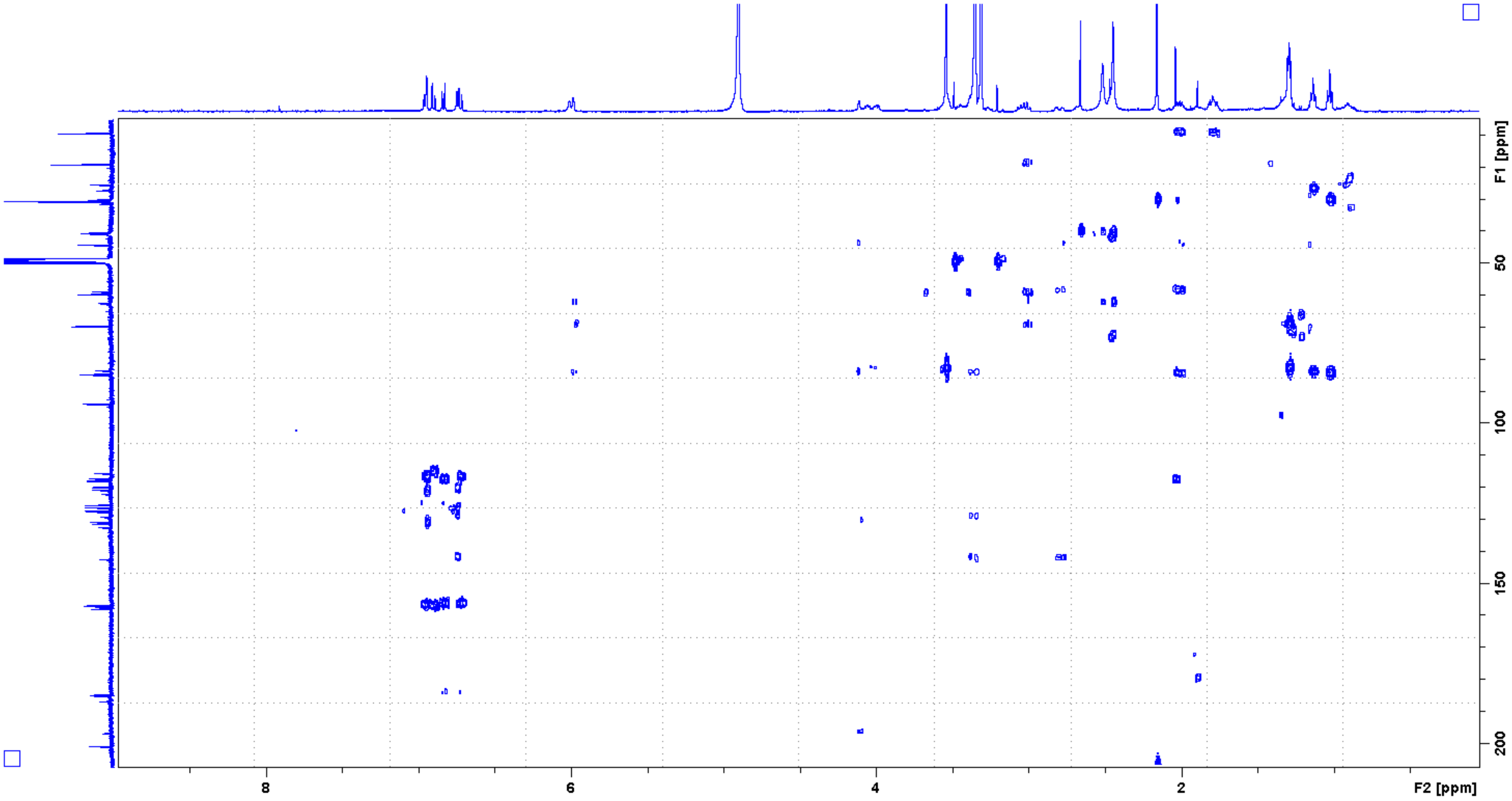

Figure S14. ROSEY Spectrum of lomaiviticin G (**2**; 500 MHz, CD_3OD)

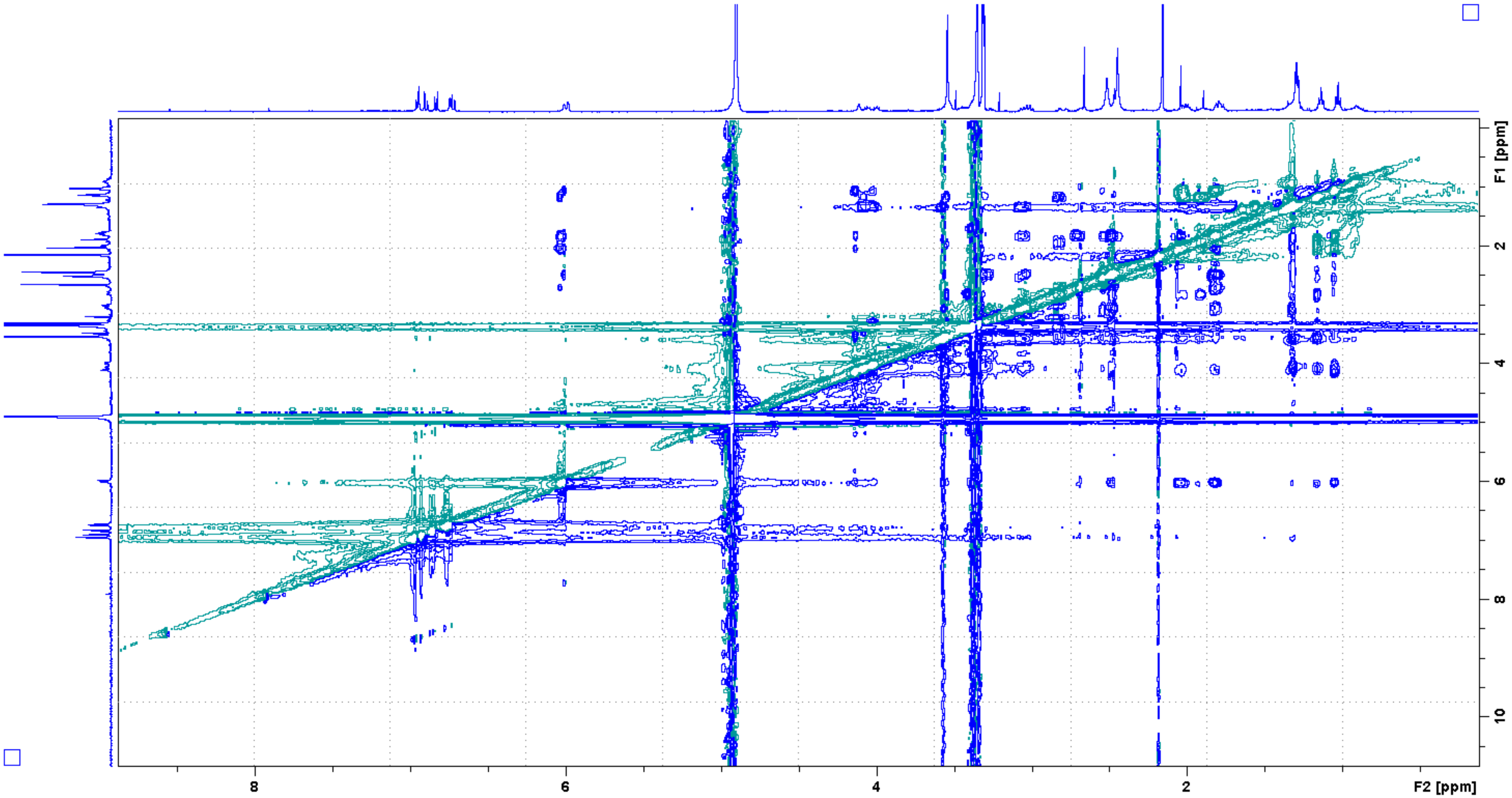

Figure S15. ^{1}H NMR spectrum of ^{13}C labeled lomaiviticin G (**2**; 500 MHz, CD_3OD)

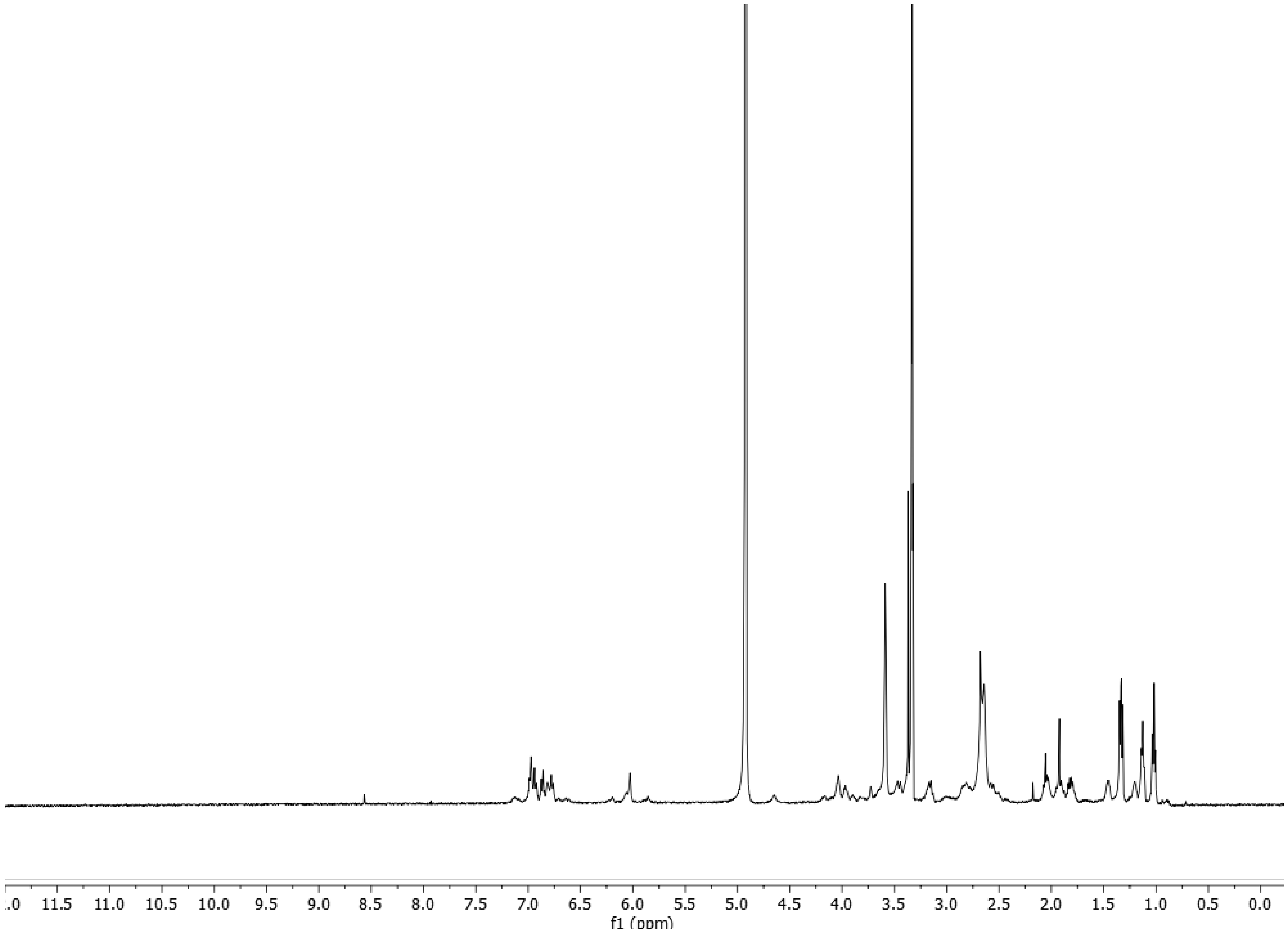

Figure S16. ^{13}C NMR spectrum of ^{13}C labeled lomaiviticin G (**2**; 500 MHz, CD_3OD)

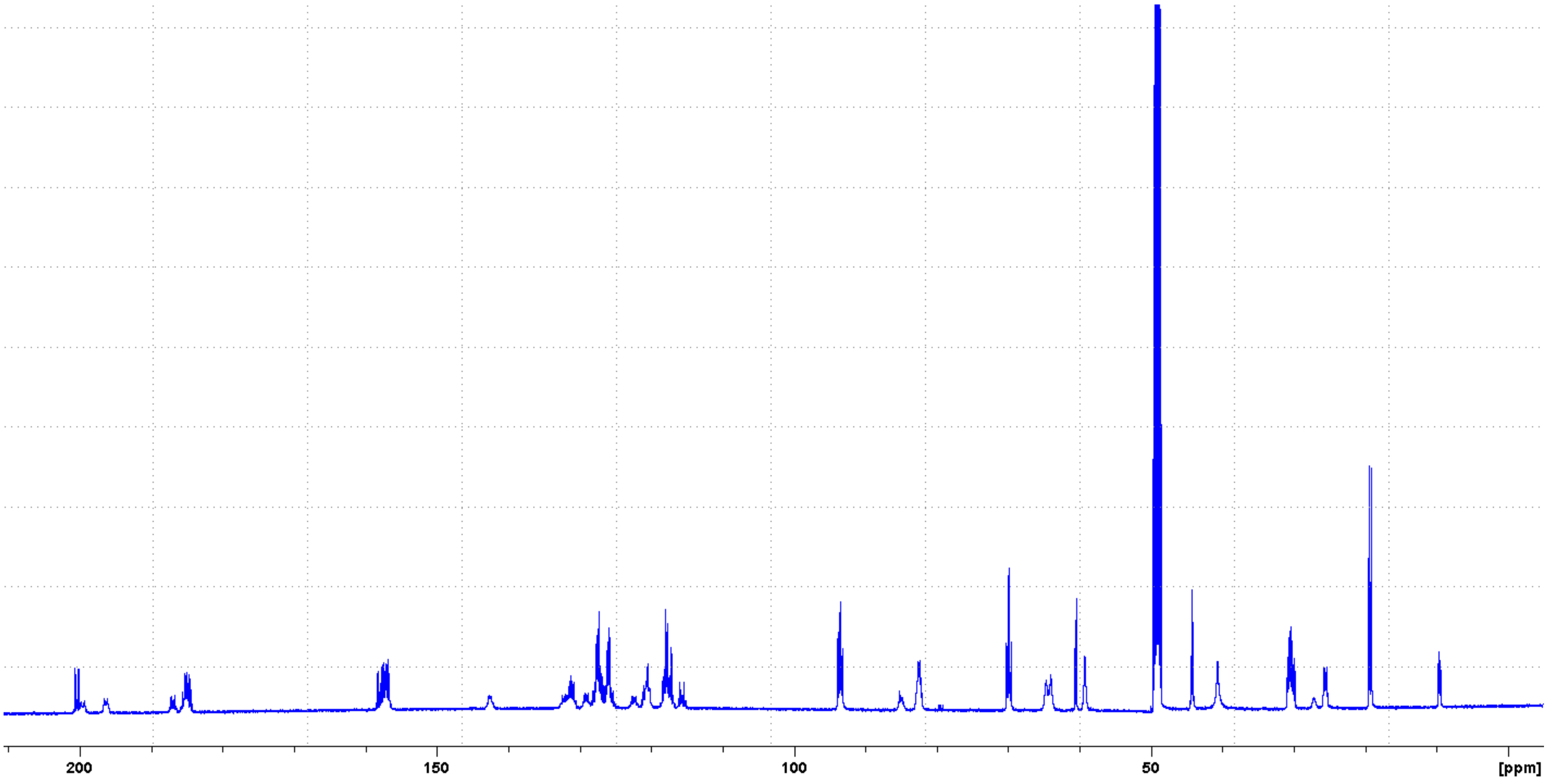

Figure S17. ^{1}H NMR spectrum comparation between ^{13}C labeled (top) and unlabeled (bottom) lomaiviticin G (**2**; 500 MHz, CD_3OD)

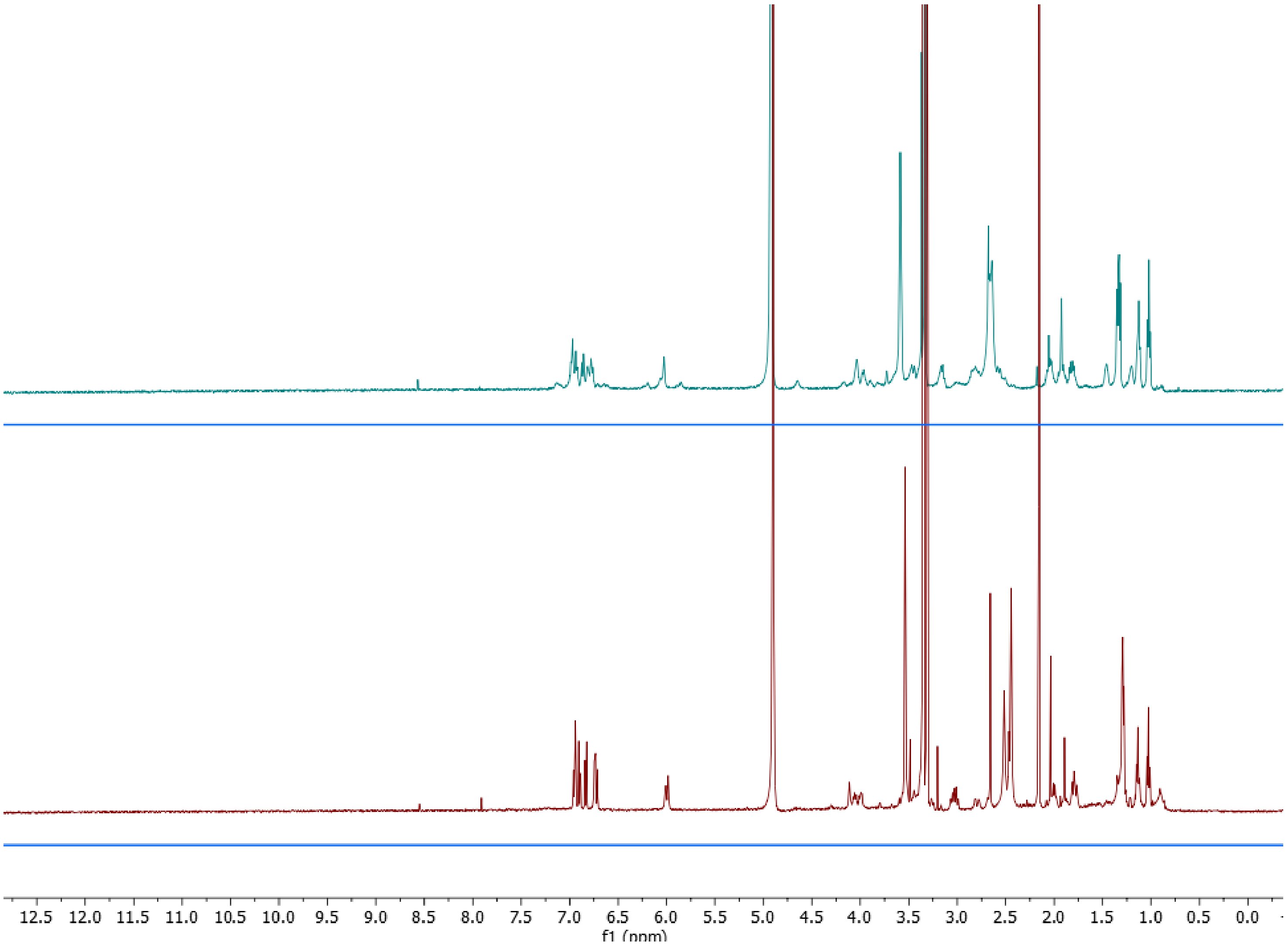

Figure S18. ^{13}C NMR spectrum comparation between ^{13}C labeled (top) and unlabeled (bottom) lomaiviticin G (**2**; 125 MHz, CD_3OD)

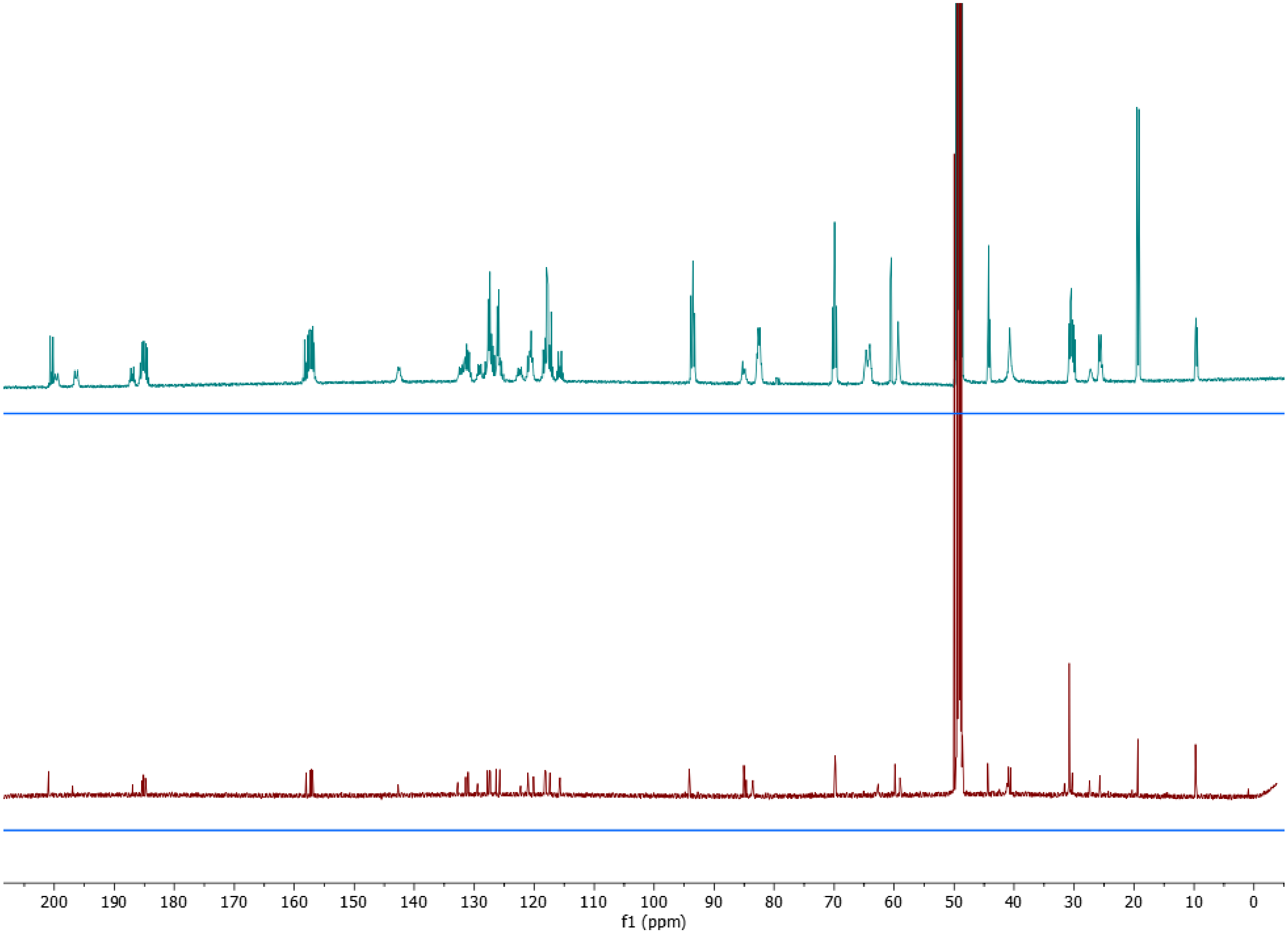

Figure S19. ^{13}C-^{13}C COSY spectrum of ^{13}C labeled lomaiviticin G (**2**; 125 MHz, CD_3OD)

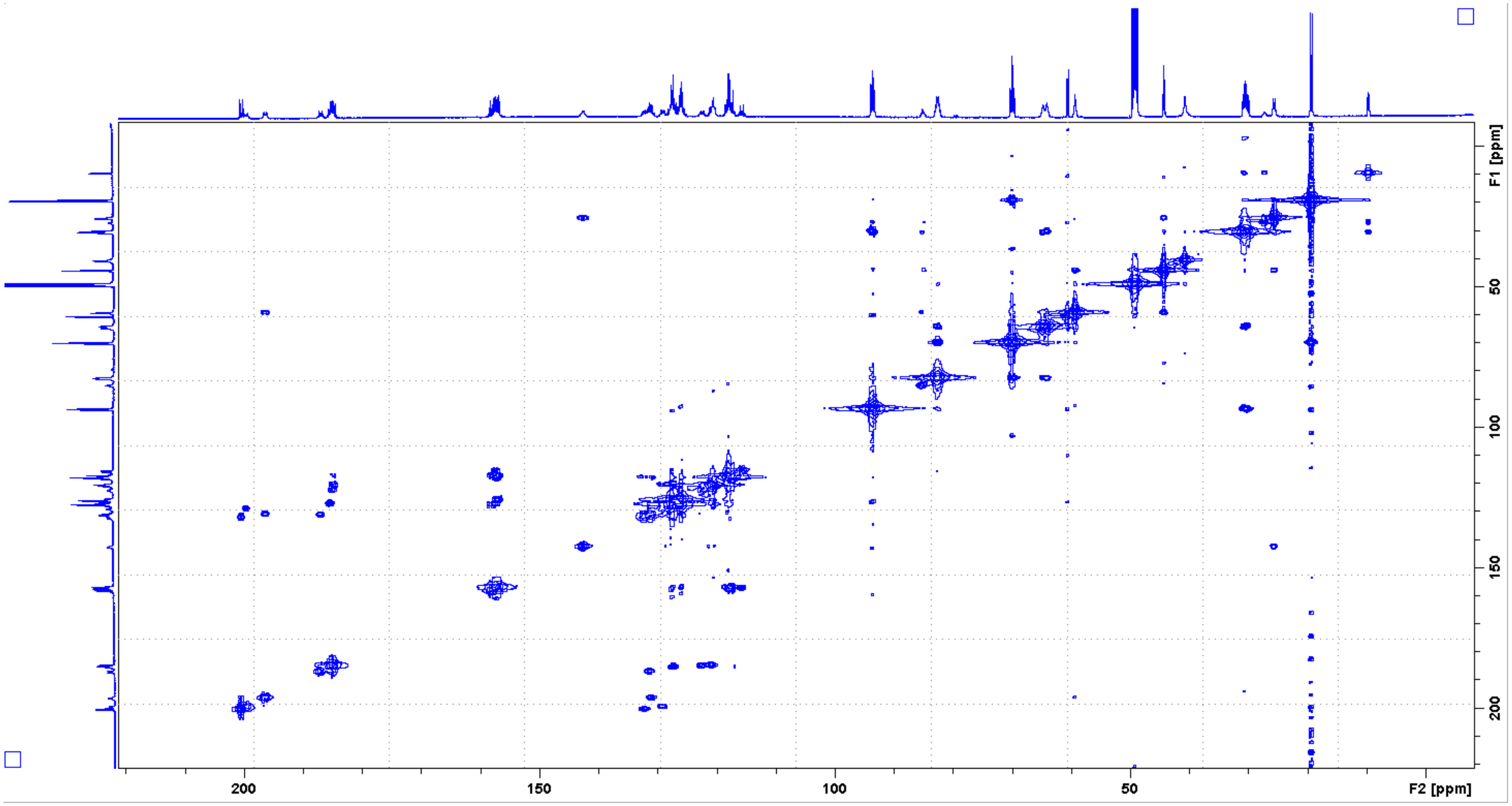

Figure S20. Positive ion HRESIMS of lomaiviticin G (**2**)

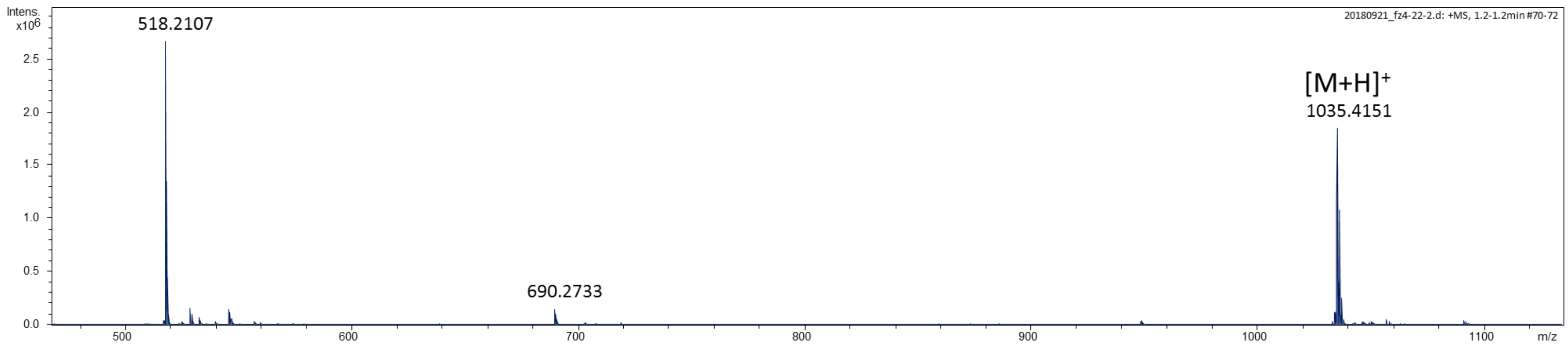

Figure S21. Positive ion ESI-MS/MS spectrum of lomaiviticin G (**2**)

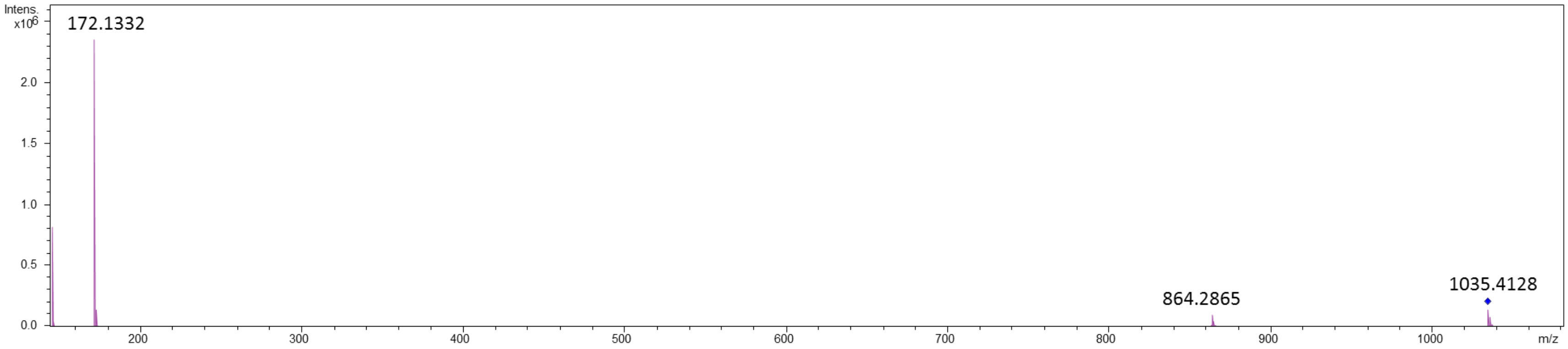

Figure S22. ^{1}H NMR spectrum of lomaiviticin H (**3**; 500 MHz, CD_3OD)

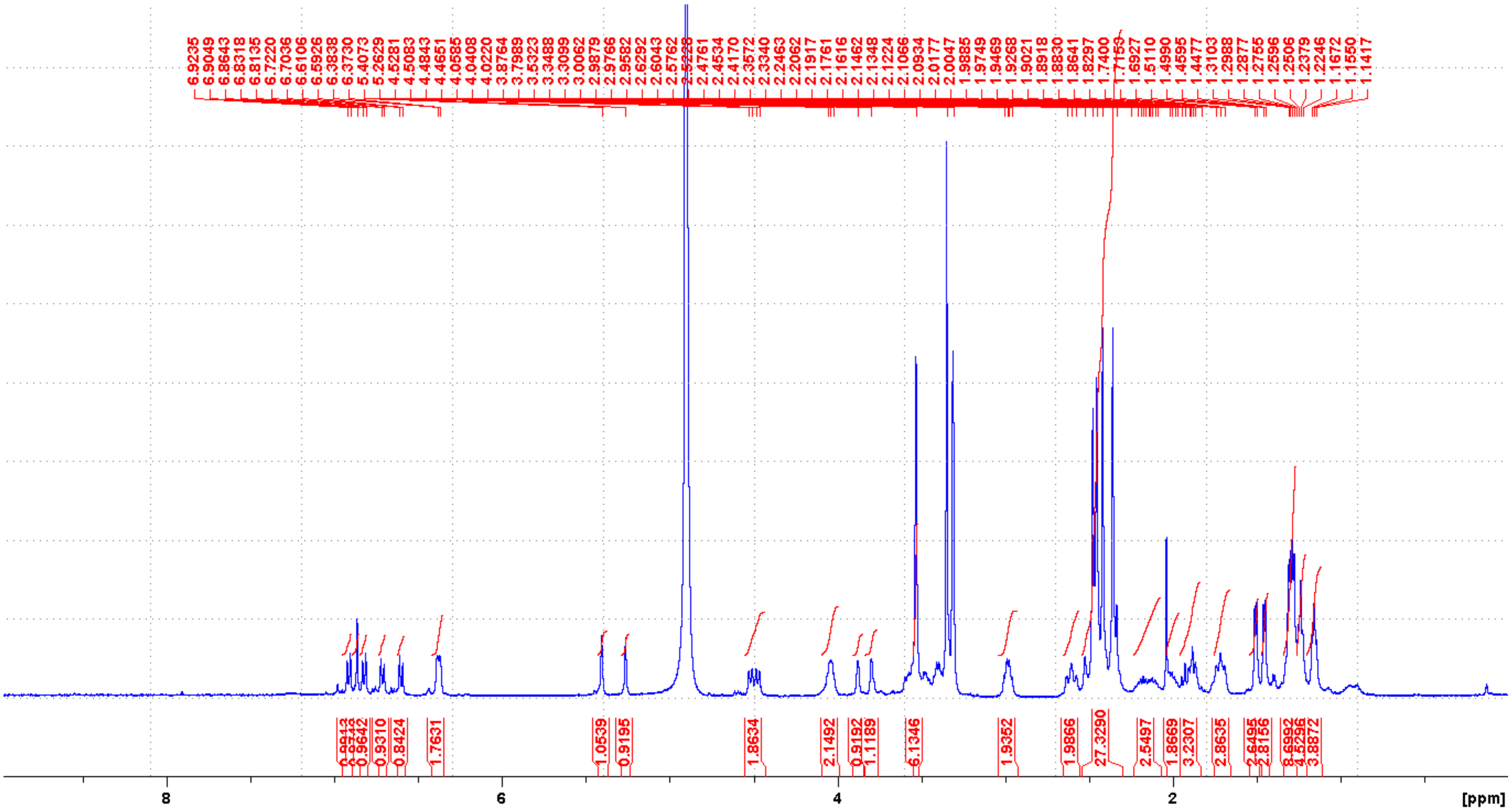

Figure S23. ^{13}C NMR spectrum of lomaiviticin H (**3**; 125 MHz, CD_3OD)

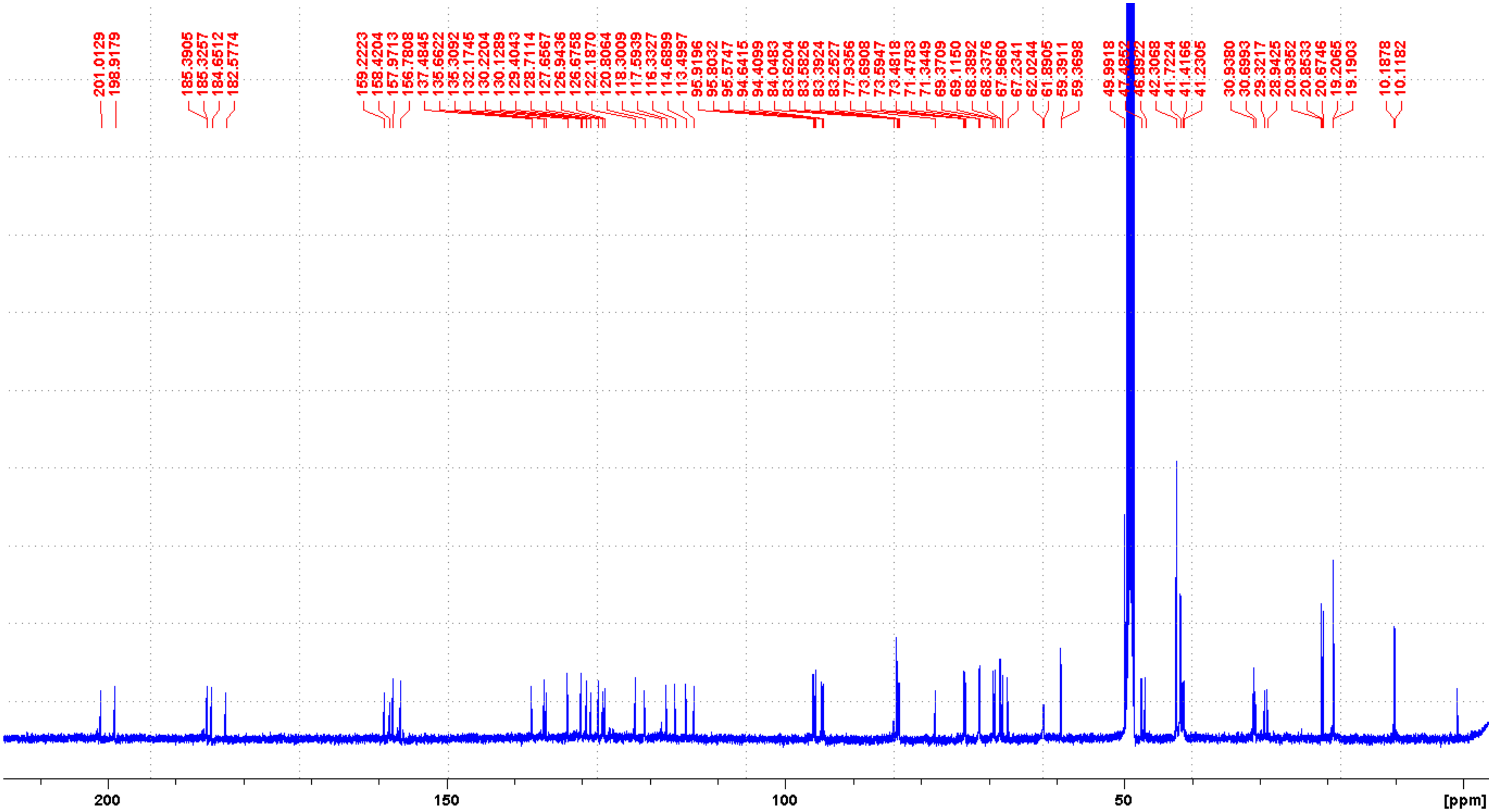

Figure S24. gCOSY spectrum of lomaiviticin H (**3**; 500 MHz, CD_3OD)

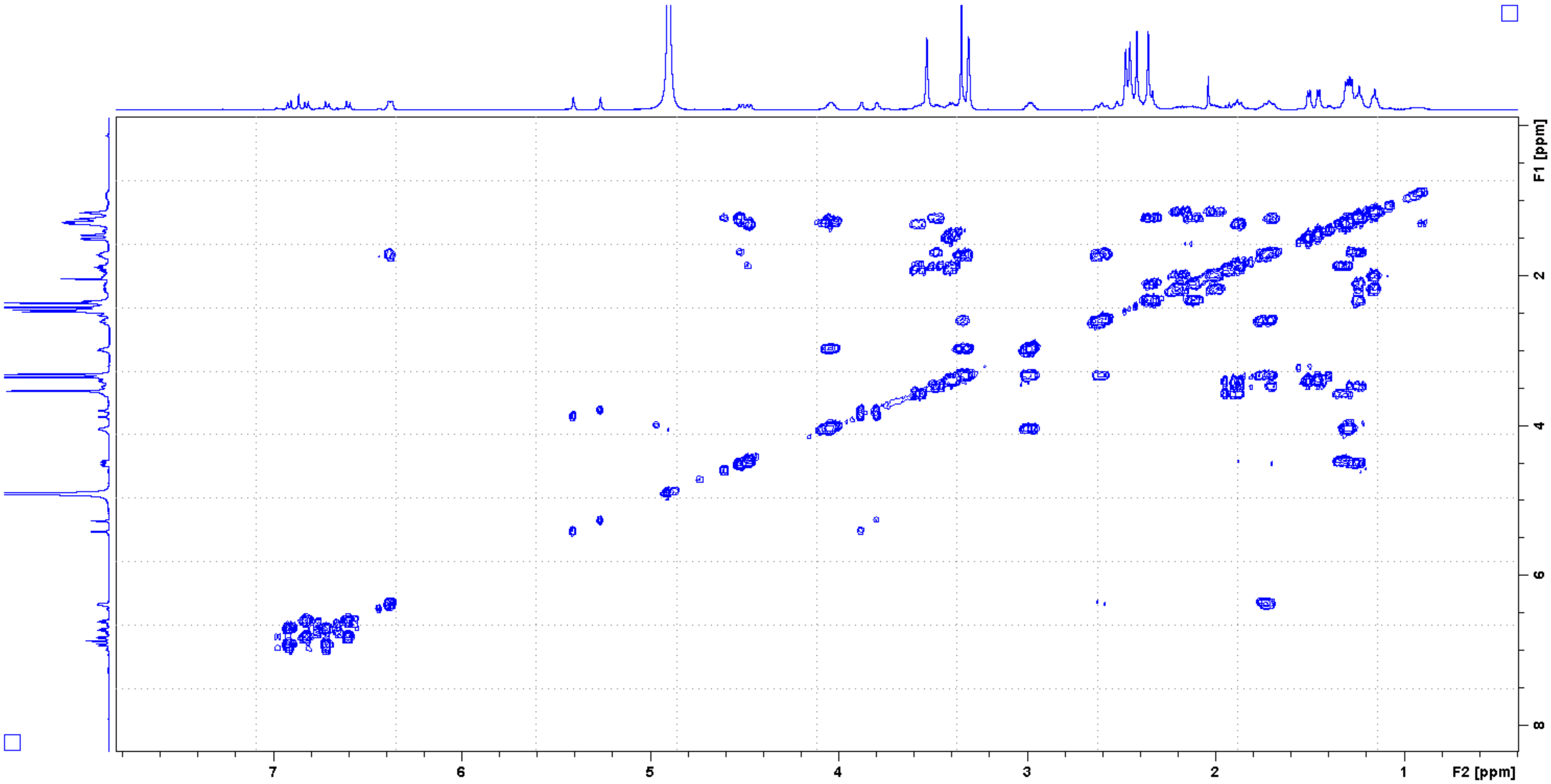

Figure S25. gHSQC spectrum of lomaiviticin H (**3**; 500 MHz, CD_3OD)

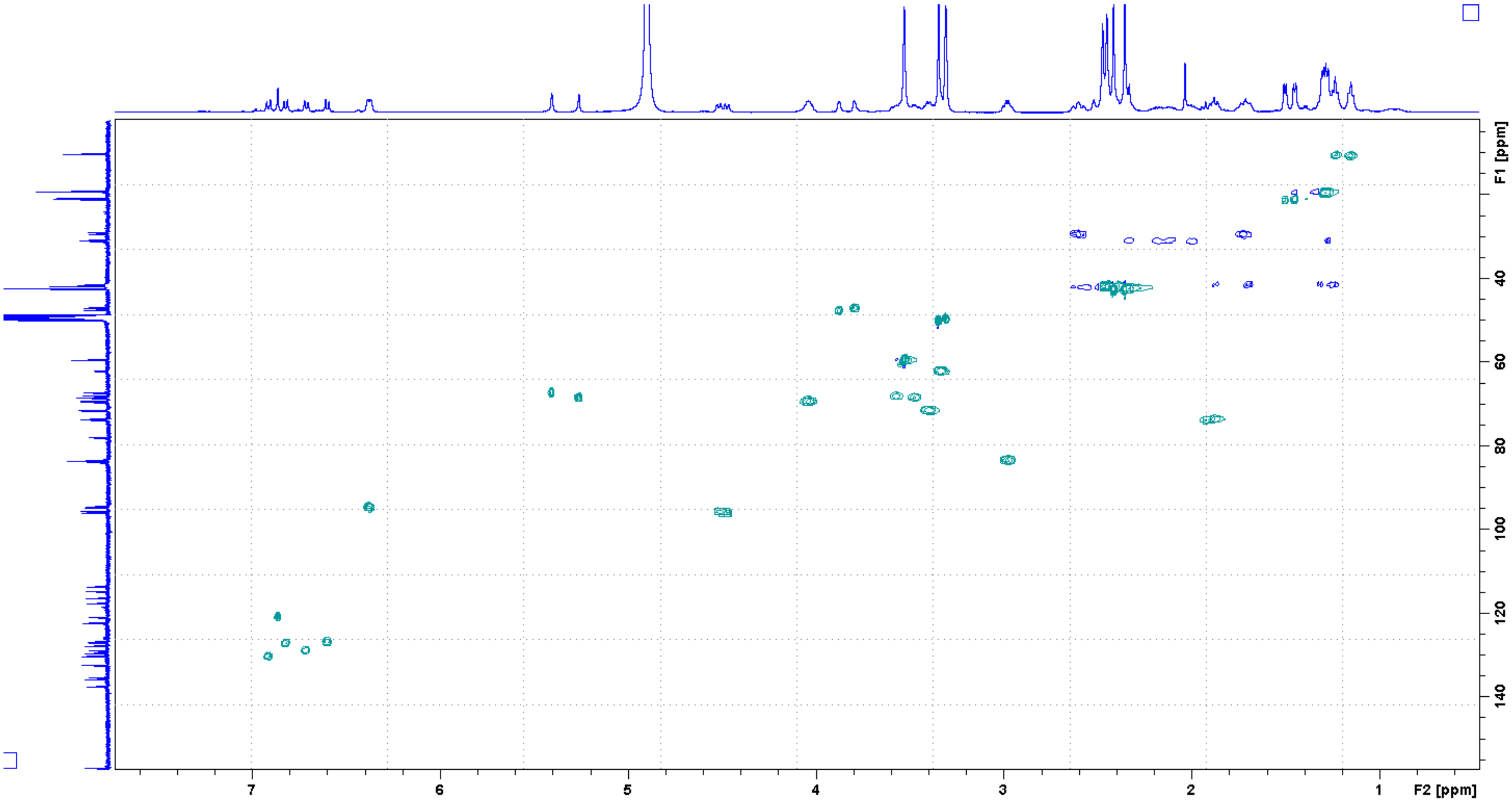

Figure S26. gHMBC spectrum of lomaiviticin H (**3**; 500 MHz, CD_3OD)

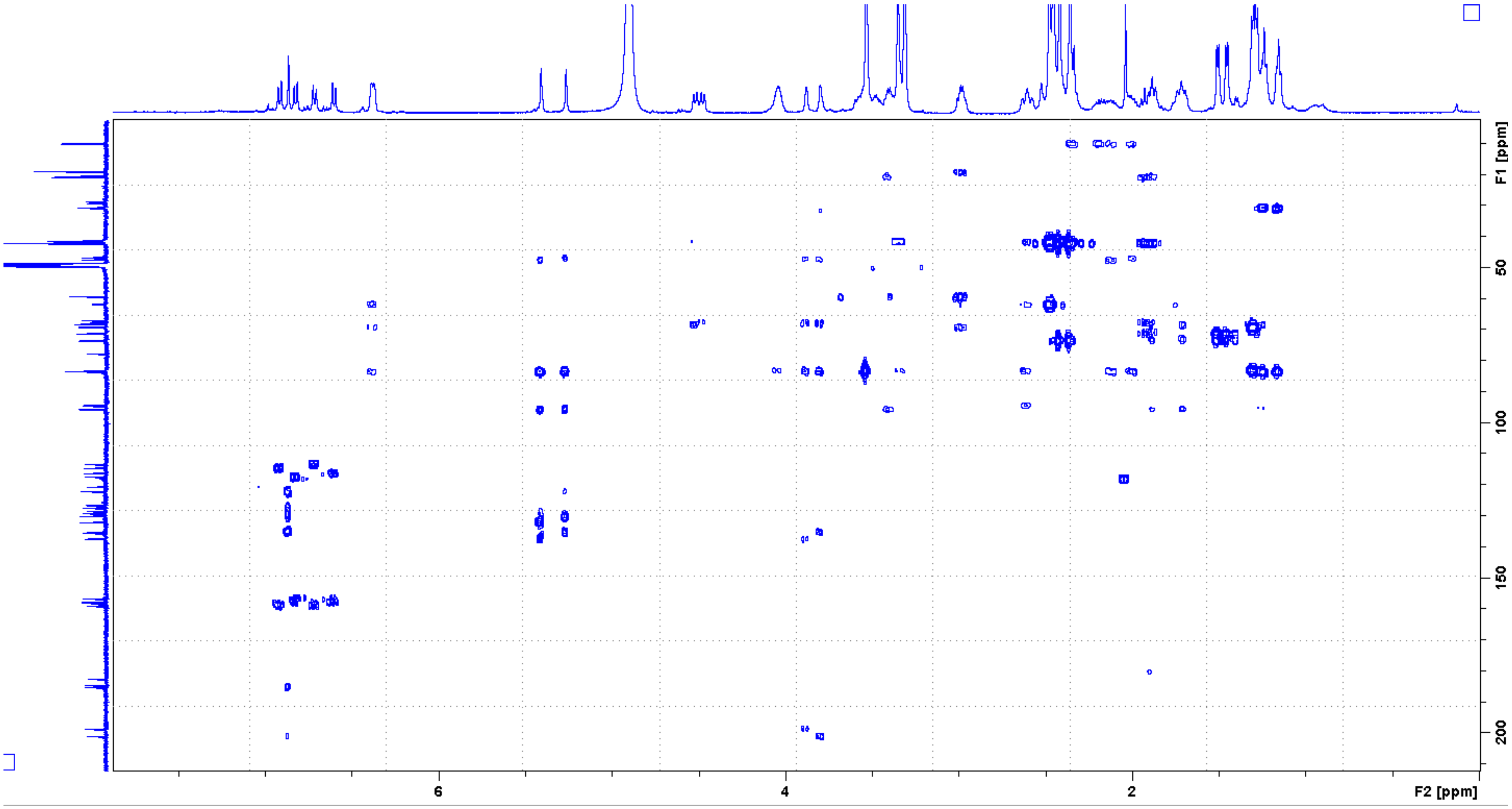

Figure S27. ROSEY Spectrum of lomaiviticin H (**3**; 500 MHz, CD_3OD)

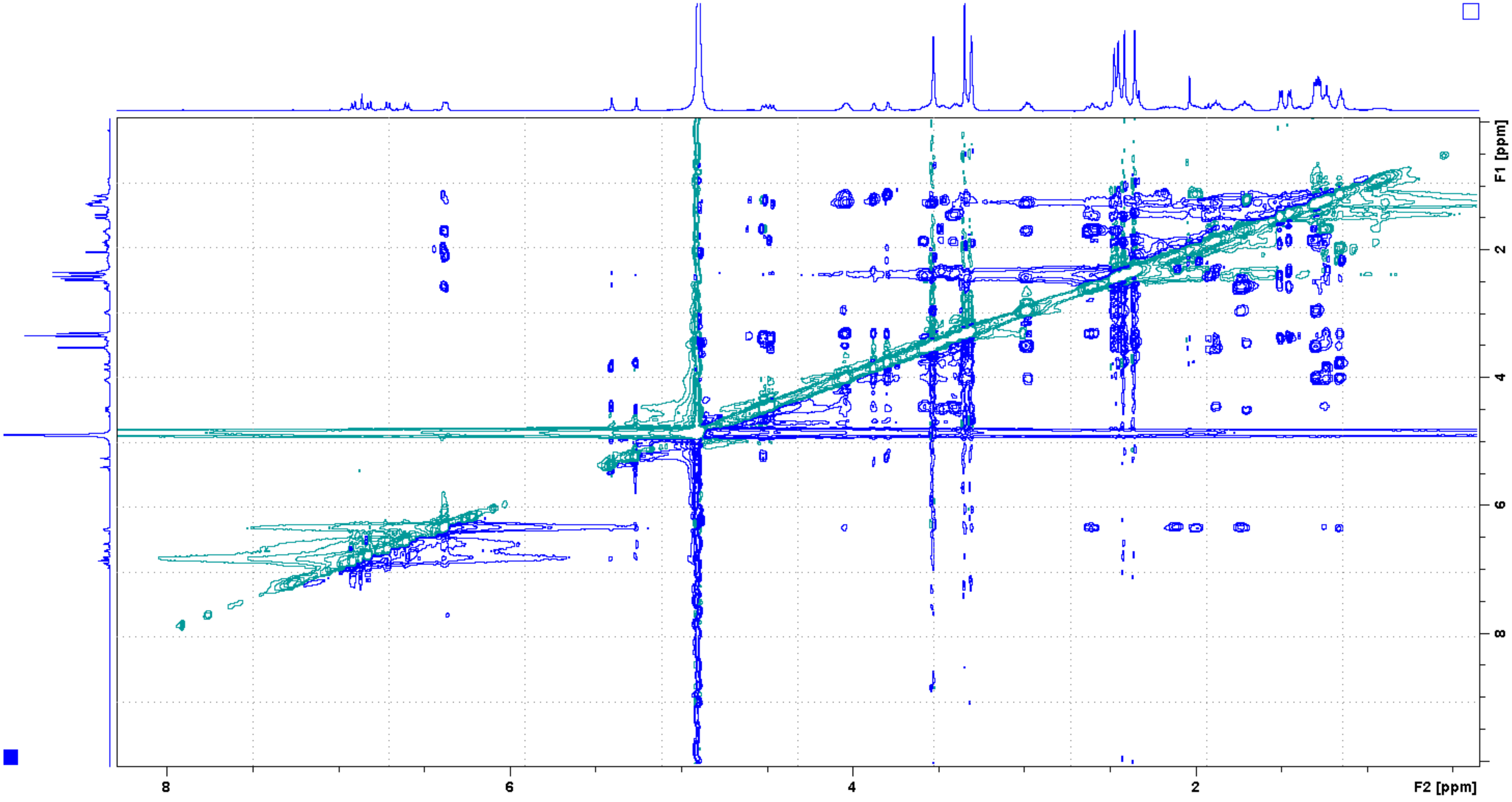

Figure S28. Positive ion HRESIMS of lomaiviticin H (**3**)

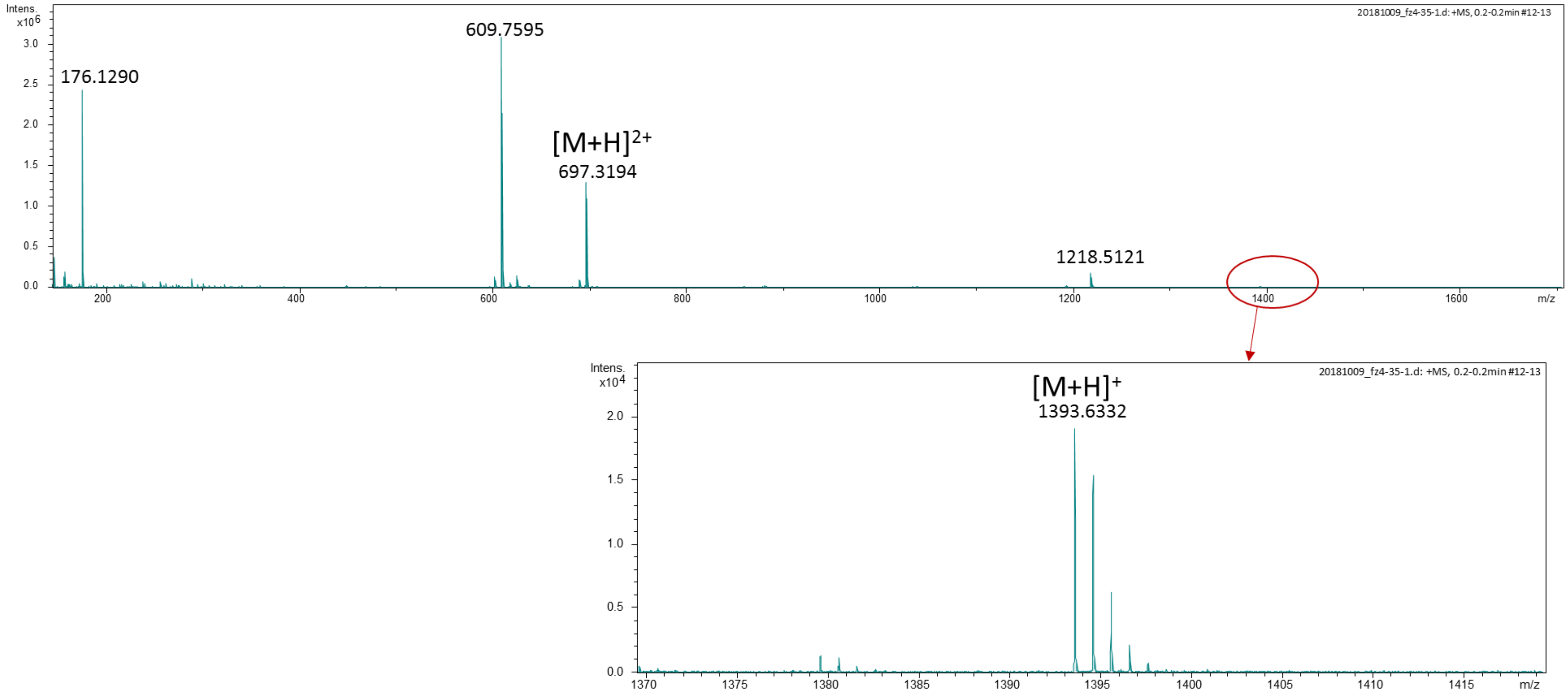

Figure S29. Positive ion ESI-MS/MS spectrum of lomaiviticin H (**3**)

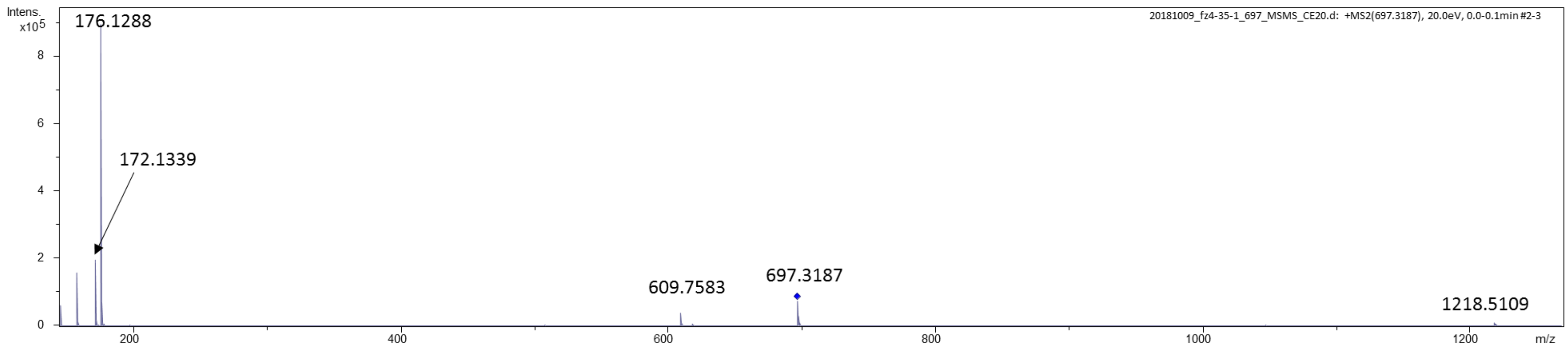

Figure S30. CD spectra of **1–3**

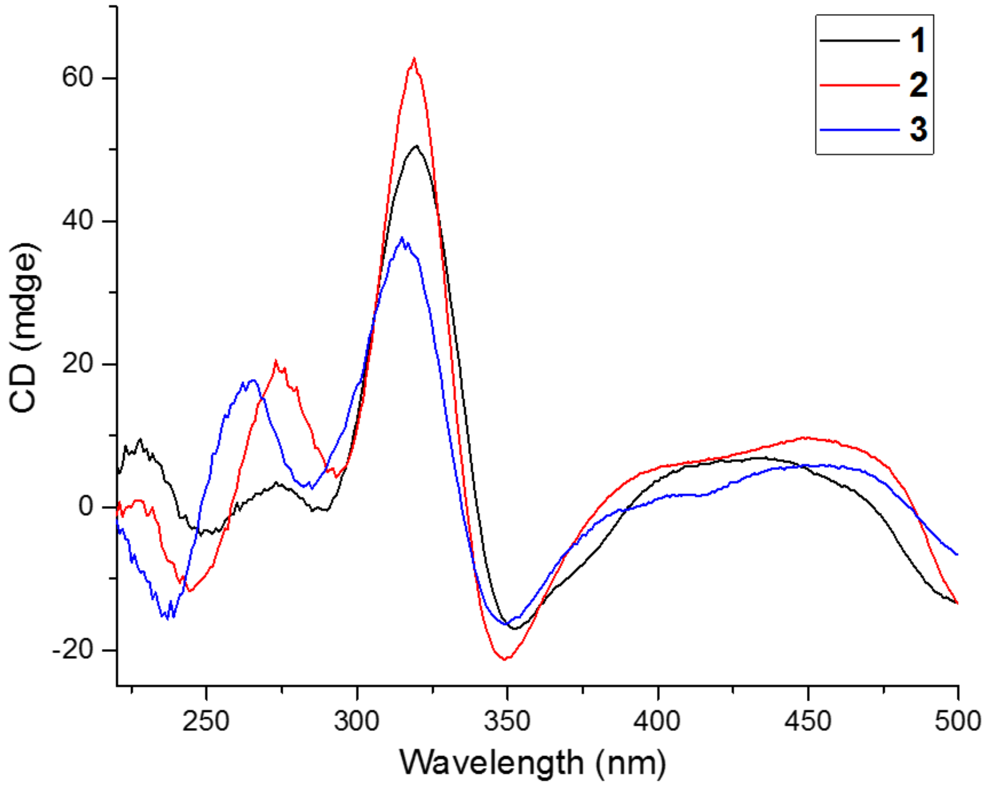

Supplement: Supplementary file 1 [file marinedrugs-23-00065-s001.zip › marinedrugs-3453480-supplementary.pdf]
